# Arabidopsis Lunapark proteins are involved in ER cisternae formation

**DOI:** 10.1111/nph.15228

**Published:** 2018-05-25

**Authors:** Verena Kriechbaumer, Emily Breeze, Charlotte Pain, Frances Tolmie, Lorenzo Frigerio, Chris Hawes

**Affiliations:** ^1^ Plant Cell Biology, Biological and Medical Sciences Oxford Brookes University Oxford OX3 0BP UK; ^2^ School of Life Sciences University of Warwick Gibbet Hill Coventry CV4 7AL UK

**Keywords:** Arabidopsis, cisternae, endoplasmic reticulum, Lunapark (LNP), network, tubules

## Abstract

- The plant endoplasmic reticulum (ER) is crucial to the maintenance of cellular homeostasis. The ER consists of a dynamic and continuously remodelling network of tubules and cisternae. Several conserved membrane proteins have been implicated in formation and maintenance of the ER network in plants, such as RHD3 and the reticulon proteins. Despite the recent work in mammalian and yeast cells, the detailed molecular mechanisms of ER network organization in plants remain largely unknown. Recently, novel ER network‐shaping proteins called Lunapark (LNP) have been identified in yeast and mammalian cells.
- Here we identify two Arabidopsis LNP homologues and investigate their subcellular localization via confocal microscopy and potential function in shaping the ER network using protein–protein interaction assays and mutant analysis.
- We show that AtLNP1 overexpression in tobacco leaf epidermal cells mainly labels cisternae in the ER network, whereas AtLNP2 labels the whole ER. Overexpression of LNP proteins results in an increased abundance of ER cisternae and *lnp1* and *lnp1lnp2* amiRNA lines display a reduction in cisternae and larger polygonal areas.
- Thus, we hypothesize that AtLNP1 and AtLNP2 are involved in determining the network morphology of the plant ER, possibly by regulating the formation of ER cisternae.

## Introduction

As the first biosynthetic organelle in the plant secretory pathway, the endoplasmic reticulum (ER) underpins the production, folding and quality control of proteins (Brandizzi *et al*., 2003; Hawes *et al*., 2015), as well as lipid biosynthesis (Wallis & Browse, 2010). In addition, it also has many other functions, such as calcium homeostasis (Hong *et al*., 1999), oil and protein body formation (Huang, 1996; Herman, 2008), rubber particle formation (Brown *et al*., 2017) and auxin regulation (Friml & Jones, 2010; Kriechbaumer *et al*., 2015).

In plant cells, the ER consists of a dynamic network of cisternae (sheets) and, more predominantly, tubules which extend throughout the cytoplasm and across cellular boundaries, with intimate connections to other organelles such as the Golgi, the plasma membrane, the outer membrane of the nuclear envelope and the cytoskeleton (Goyal & Blackstone, 2013; Hawes *et al*., 2015; Stefano & Brandizzi, 2017). Hence, the ER is crucial to the maintenance of cellular homeostasis. The ER network is continually remodelling, presumably in response to differing cellular demands, with the formation of new three‐way junctions and polygons via tubule extension and fusion events, balanced with polygon loss from tubule sliding and ring closure (Griffing, 2010). Several conserved membrane proteins have been implicated in both the formation and maintenance of the ER network in plants, notably the GTPase ROOT HAIR DEFECTIVE3 (RHD3) and the reticulon (RTN) family of proteins. RHD3, orthologous to mammalian atlastins (ATL) and yeast Sey1p, may mediate membrane fusion and the formation of three‐way junctions (Chen *et al*., 2011; Zhang *et al*., 2013). These three‐way junctions of the ER consist of small triangular sheets with concave edges (Shemesh *et al*., 2014). The fusogenic action of RHD3 is then complemented by the curvature‐generating and/or stabilizing RTN proteins, which preferentially localize to ER tubules and the curved edges of cisternae. We recently demonstrated that an Arabidopsis reticulon (RTN13) relies on a conserved amphipathic helix to induce membrane curvature *in vivo* (Breeze *et al*., 2016). Indeed, purified yeast and mammalian orthologues of these two groups of proteins (Sey1p or ATL with RTNs) are sufficient to reconstitute a dynamic tubular ER network in proteoliposomes in the presence of GTP (Powers *et al*., 2017).

A third class of conserved ER network‐shaping proteins called Lunapark (Lnp1p (yeast) and mLnp1 (mammals)) has additionally been identified in yeast and mammalian cells. Lunapark (LNP) proteins are characterized by the presence of two N‐terminal transmembrane domains (TMDs) and an atypical Cys4 type C‐terminal zinc finger motif which, in yeast, mediates homodimerization and is required for LNP function (Casey *et al*., 2015; Wang *et al*., 2016). Immediately adjacent to the zinc finger lies the amino acid sequence LNPARK or a variant thereof. Mammalian Lnp1 is dependent on *N*‐myristoylation for its localization to ER junctions and morphogenic activity. The absence of this motif in yeast Lnp1p could indicate that LNP proteins in higher organisms have evolved an additional degree of functionality and/or regulation via post‐translational lipid modifications (Moriya *et al*., 2013; Wang *et al*., 2016; Turnbull & Hemsley, 2017).

Yeast and mammalian LNP proteins preferentially localize to ER three‐way junctions *in vivo* and, although they are not required for ER network formation, it has been suggested that LNP acts to stabilize these intersections, potentially acting as temporary scaffold in nascent junctions (Chen *et al*., 2012, 2015; Wang *et al*., 2016). Immunofluorescence staining of mammalian COS‐7 cells with an anti‐mLnp1 antibody showed that only around half of all three‐way junctions contained mLnp1 but that mLnp1 acquisition was associated with enhanced junction survival probability and reduced junction mobility and ring closure (Chen *et al*., 2015).

Lnp1p has been reported to work synergistically with RTNs and Yop1 (an additional curvature stabilizing protein found in yeast, orthologous to mammalian REEPs), but in antagonism to Sey1p to maintain the cortical ER network in yeast cells (Chen *et al*., 2012). Indeed, in *sey1∆* yeast mutants lacking functional Sey1p, Lnp1p is expressed in cisternae throughout the ER network, including the nuclear envelope, suggesting that in the wild‐type, Sey1 acts upstream of Lnp1, restricting Lnp1 to ER tubule junctions (Chen *et al*., 2012). Coimmunoprecipitation of epitope‐tagged Lnp1p with RTN1p, Sey1p or Yop1p proteins showed that Lnp1p was capable of interacting with all three yeast ER morphogens *in vivo*, and that, moreover, the interaction of Lnp1p with Rtn1p is negatively regulated by Sey1p (Chen *et al*., 2012). However, more recently Wang *et al*. (2016) reported that in mammalian cells stably expressing both Lnp‐mCherry and GFP‐ATL‐3, ATL was in close proximity to Lnp‐mCherry in the peripheral ER network but, crucially, the two proteins did not precisely colocalize and no interaction was found in reciprocal pull‐down experiments.

Despite the recent work in mammalian and yeast cells, the detailed molecular mechanisms of ER network organization in plants remain largely unknown. Here we identify two Arabidopsis LNP homologues and show that their overexpression (as fluorescent protein fusions) in tobacco leaf epidermal cells results in the proteins labelling the ER and accumulating at cisternae and a small proportion of three‐way junctions of the ER network. Furthermore, overexpression of LNP proteins results in an increased abundance of cisternae in the ER network. Thus, we hypothesize that AtLNP1 and AtLNP2 are involved in determining the dynamic morphology of the plant ER, possibly by regulating the formation of ER cisternae.

## Materials and Methods

### Comparative genomics

Genes orthologous to LNP1 and LNP2 in other species within the land plant lineage (Embryophyta) and containing the InterPro LNP domain (IPR019273) were identified using the Plaza 4.0 online platform (Proost *et al*., 2009). This resulted in the identification of 87 LNP‐like genes from 49 plant species. A phylogenetic tree of the homologous LNP gene family was assembled within Plaza, alongside a multiple sequence alignment of the amino acid sequences. A consensus sequence logo of the LNPRK amino acid motif from the multiple sequence alignment was generated using WebLogo3 (Crooks *et al*., 2004).

### Cloning of expression plasmids

Primers were obtained from Eurofins Genomics (Ebersberg, Germany). Q5 high‐fidelity DNA polymerase (New England Biolabs, Ipswich, MA, USA) was used for all PCR reactions. Genes of interest were cloned with a C‐terminal GFP fusion under a ubiquitin‐10 promoter (*P*_*UBQ10*_) (Grefen *et al*., 2010) using Gateway technology (Invitrogen).

The *LNP1* and *LNP2* promoter and coding sequences were amplified from Col‐0 genomic DNA using the gene‐specific primers LP: TTCAAACAATTACAAACTTAACGGTAGC and RP: GTTTGGTGTCTCATTCTCAGCTGTTTCC for LNP1 and LP: CCAGCTTGTGTGAATATGGTTTGAGCTT and RP: GCTCGGTGTCCCGGTCTCAGTAATTGC for LNP2 and cloned into the pDONR/Zeo Gateway Entry vector. Thus, these *LNP1* and *LNP2* constructs contain 1910 and 2090 bp, respectively, upstream of the ATG start site, and terminate immediately before the TGA stop sites.

### Preparation of *lnp1* and *lnp1lnp2* amiRNA lines

Candidate amiRNA sequences specific to both *LNP1* and *LNP2* coding regions were identified using the Web MicroRNA Designer (WMD) platform (http://wmd3.weigelworld.org) (Schwab *et al*., 2006; Ossowski *et al*., 2008). Two amiRNA sequences were selected (targeted against the region immediately downstream of TMD2 (amiRNA1) or the conserved zinc finger domain (amiRNA2); Supporting Information Fig. S1) and cloned into the naturally occurring Arabidopsis miR319a replacing the target‐specific sequence using a series of overlapping PCRs (as described by Ossowski *et al*., 2008) and with the addition of Gateway‐compatible attB sites. The purified attB‐amiRNA precursors were subsequently used to generate Entry (pDONR/Zeo) and 35S destination (pB7WG2) clones. The 35S constructs were transformed by heat shock into *Agrobacterium tumefaciens* strain GV3101 and stable homozygous Arabidopsis lines created via the floral dip procedure (Clough & Bent, 1998). RNA was extracted from dry seeds of two independent amiRNA1‐ and amiRNA2‐containing lines and Col‐0 as described by Meng & Feldman (2010) and first‐strand cDNA synthesized using ReadyScript (Sigma Aldrich) according to the manufacturer's instructions. Semiquantitative reverse transcription polymerase chain reaction was performed using primers specific to LNP1 (LP: TGCCAAACACTCGGGAGG and RP: AGGGATTGGACTGTTACCGC) and LNP2 (LP: GAGCAATGACATGGAGGTTAAC and RP: GTCTCAATCAGTGGCAGAGAG) (Fig. S1), together with At4g34270 (LP: GTGAAAACTGTTGGAGAGAAGCAA and RP: TCAACTGGATACCCTTTCGCA) and At4g12590 (LP: GAGATGAAAATGCCATTGATGAC and RP: GCACCCAGACTCTTTGATG) (seed‐specific housekeeping reference genes (Dekkers *et al*., 2012)) as loading controls, and expression levels in the amiRNA mutants compared with that of wild‐type, Col‐0 (Fig. S1). The amiRNA1‐containing lines were found to have reduced expression of *LNP1* but comparable expression of *LNP2* to that detected in the wild‐type and was subsequently referred to as *lnp1*, whereas the amiRNA2‐containing lines had markedly reduced expression levels of both *LNP1* and *LNP2* and were therefore denoted as *lnp1lnp2*. The differing specificity of the two amiRNAs for the *LNP* targets is likely to be the result of nucleotide mismatches between the two target sequences, notably in the crucial 5′ portion of the amiRNA (positions 2–12) (Schwab *et al*., 2006). Design of amiRNAs to successfully silence a specific gene target is challenging given the absence of canonical miRNA sequence parameters.

### Plant material and transient expression in tobacco leaves

For Agrobacterium‐mediated transient expression, 5‐wk‐old tobacco (*Nicotiana tabacum* SR1 cv Petit Havana) plants grown in the glasshouse were used. Transient expression was carried out according to Sparkes *et al*. (2006). In brief, each construct was introduced into Agrobacterium strain GV3101 by heat shock. Transformants were inoculated into 3 ml of YEB medium (l^–1^: 5 g of beef extract, 1 g of yeast extract, 5 g of sucrose and 0.5 g of MgSO_4_ 7H_2_O) with 50 μg ml^−1^ spectinomycin and 25 μg ml^−1^ rifampicin. After overnight shaking at 25°C, 1 ml of the bacterial culture was pelleted by centrifugation at 2200 ***g*** for 5 min at room temperature. The pellet was washed twice with 1 ml of infiltration buffer (50 mM MES, 2 mM Na_3_PO_4_ 12H_2_O, 0.1 mM acetosyringone and 5 mg ml^−1^ glucose) and then resuspended in 1 ml of infiltration buffer. The bacterial suspension was diluted to a final OD_600_ of 0.05 (0.01–0.3 in the OD_600_ serial dilution series) and carefully pressed through the stomata on the lower epidermal surface using a 1 ml syringe. Infiltrated plants were then returned to glasshouse conditions for 48 h before imaging.

### Confocal microscopy

Images were taken using a Zeiss 880 laser scanning confocal microscope with ×100/1.46 numerical aperture DIC M27 Elyra oil immersion objective. For imaging of the green/red fluorescent protein (GFP/RFP) combinations, samples were excited using 488 and 561 nm laser lines in multitrack mode with line switching. Signals were collected using the high‐resolution Airyscan detector (Zeiss, Oberkochen, Germany) with emission wavelength of 523 nm for GFP and 579 nm for RFP. Images were edited using the Zen image browser (Zeiss).

### Lipid dye Rhodamine B hexyl ester

Staining the ER with Rhodamine B hexyl ester was carried out according to Hawes *et al*. (2018). Rhodamine B hexyl ester solution was prepared as a 1 mM stock solution in dimethyl sulphoxide and a 1 μM working solution in distilled water (DW). Whole Arabidopsis seedlings 7–10 d after germination were transferred to Eppendorf tubes containing 1 μM Rhodamine B hexyl ester. Seedlings were incubated for 15 min in the dye and washed in DW. Samples were imaged with a 514 nm argon ion laser emission detected using 470–500 and 560–615 nm bandpass filters.

### FRET‐FLIM data acquisition

Constructs were transiently expressed in tobacco leaf epidermal cells as described earlier. Leaf discs were excised and the GFP and mRFP expression levels in the plant within the region of interest were confirmed using a Nikon EC2 confocal microscope at 488 and 543 nm, respectively. Förster resonance energy transfer by fluorescence lifetime imaging microscopy (FRET‐FLIM) data capture was performed according to Kriechbaumer *et al*. (2015) using a two‐photon microscope at the Central Laser Facility of the Rutherford Appleton Laboratory. A two‐photon microscope built around a Nikon TE2000‐U inverted microscope was used with a modified Nikon EC2 confocal scanning microscope to allow for multiphoton FLIM (Schoberer & Botchway, 2014). At least three nuclei (*n*_(RTN1‐RTN1)_ = 3; *n*_(RTN1‐LNP1)_ = 10; *n*_(RTN1‐LNP2)_ = 12) from at least two independent biological samples per protein–protein combination were analysed, and the average of the ranges was taken.

### Quantification of ER structure parameters in WT and mutants

**Figure 1:**
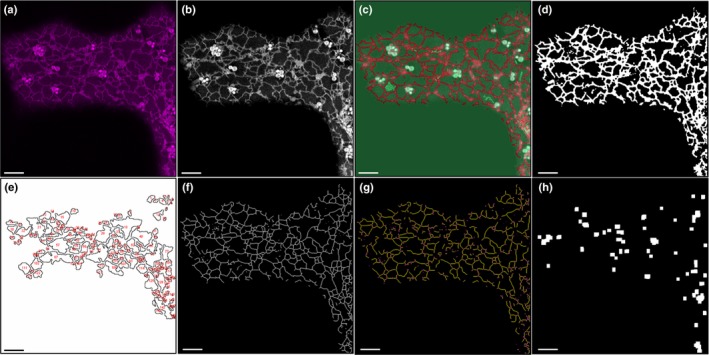
Endoplasmic reticulum (ER) network analysis workflow for Arabidopsis *lnp1* and *lnp1lnp2* amiRNA mutants. The ER network was analysed and quantified using the following steps. Example images are shown for every step. Bars, 5 μm. (a) Initial image; (b) enhanced contrast; (c) Weka trainable segmentation: red regions correspond to detected ER structures, green highlights rejected ER regions (e.g. here for *lnp1* mutant stained mitochondria); (d) binary image of segmented ER; (e) polygonal region analysis output with numbered outlines of each polygonal region that is fully enclosed by the ER; (f) skeletonized ER structure; (g) analysed ER skeleton with blue pixels indicating detected end points (single pixel wide) and magenta pixels showing detected junctions; (h) cisternae detected by an iterative opening function.

Images acquired with a Zeiss 880 confocal microscope with Airyscan (see methods confocal microscopy) were analysed using ImageJ (National Institutes of Health, Bethesda, MD, USA; Fig. 1). Confocal images were imported to ImageJ and smoothed in order to reduce noise. The ER signal was segmented from the background and mitochondrial signal using the ImageJ plugin Trainable Weka Segmentation. Once segmented, a closing function was applied to reduce segmentation errors and to ensure high connectivity of the network. To analyse the polygonal regions, a region of interest was drawn around the cell using a digitizing tablet, and the ImageJ ‘Analyse particles’ command was applied. Only polygonal regions completely enclosed by the network were considered for the analysis. As the data are not normal, the Wilcoxon rank sum test was applied. *P* < 1.7282e^−17^ (*lnp1*) and *P* < 2.8930e^−44^ (*lnp1lnp2*) were calculated for three biological replicas with at least 12 technical repeats each. To analyse ER network structure, an opening function with several iterations was applied to the segmented ER image to isolate cisternae. The total area of the cisternae was measured and the percentage of the cell surface classified as cisternae was calculated (*P* < 6.0051e^−4^ in *lnp1* and *P* < 0.0031 for in *lnp1lnp2*). The segmented ER network was skeletononized and the cisternae identified in the previous step were subtracted to produce a skeleton of the tubular ER network. Using the ‘Analyse skeleton’ ImageJ command, all three‐way junctions in the skeletonized tubular network were identified. The number of three‐way junctions identified was normalized against the area of the cell surface (*P* > 0.2643 for *lnp1* and *P* > 0.7427).

### Persistency analysis

To analyse persistency of LNP1‐labelled punctae and cisternae, videos were taken using the Zeiss 880 confocal with Airyscan. RFP‐HDEL was used as an ER luminal marker. The ImageJ plugin ‘temporal color‐code’ was applied to colour‐code movement over time; in this case, in three image frames at 0, 30 and 60 s. White areas indicate points of persistency throughout in the composite image, and red areas indicate highest mobility.

### Accession numbers

AtLNP1, At2g24330.1; AtLNP2, At4g31080.1; AtLNP2.2, At4g31080.2.

## Results

### Identification of two LNP orthologues in Arabidopsis

**Figure 2:**
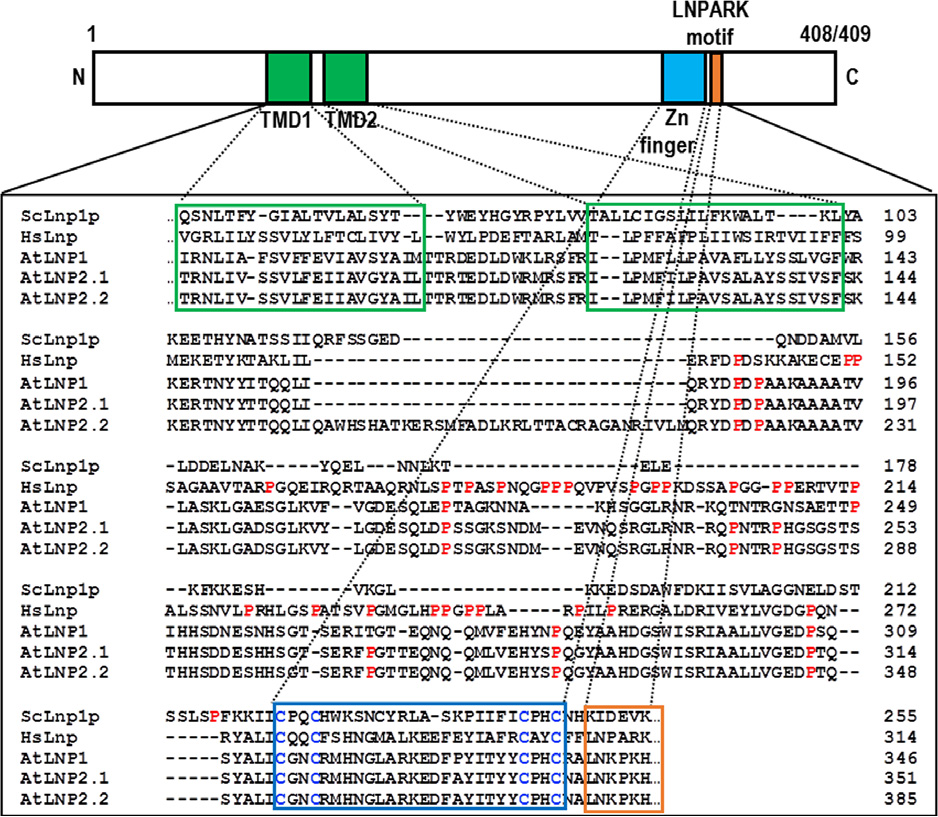
AtLNP1 and AtLNP2 are orthologous to Lunapark proteins in other organisms. Two putative Lunapark (LNP) proteins were identified in *Arabidopsis thaliana*, with sequence homology and conserved motifs (two transmembrane domains, TMDs; zinc finger, and LNPARK motif (LNKPKH in Arabidopsis)) with previously annotated *Homo sapiens* Lnp (HsLnp) and *Saccharomyces cerevisiae* Lnp1p (ScLnp1p) proteins. Note that unlike yeast and Arabidopsis LNP proteins, HsLnp contains an additional Pro‐rich region (Pro residues are shown in red). AtLNP1 and AtLNP2.1 are 408 and 409 amino acid residues in length, respectively.

The protein sequences of yeast Lnp1p and human Lnp were queried against the *Arabidopsis thaliana* proteome using Blastp (Altschul *et al*., 1990). This analysis identified two LNP orthologues, At2g24330 and At4g31080 (subsequently named AtLNP1 and AtLNP2, respectively), both with *c*. 24% amino acid identity to the queried sequences across the entire length of the protein. AtLNP1 and 2 themselves share 67% amino acid identity. According to large‐scale microarray data in the eFP browser (Winter *et al*., 2007) both At*LNP1* and At*LNP2* are transcribed ubiquitously but At*LNP2* has increased transcription abundance in pollen (Fig. S2). Further comparison of the identified AtLNP protein sequences with their mammalian and yeast orthologues revealed the presence of several conserved features, notably two TMDs towards the N‐termini and a zinc finger motif immediately adjacent to the LNPARK motif (Fig. 2). However, unlike the human (and mouse) Lnp proteins, AtLNP1 and 2 do not contain a Pro‐rich domain upstream of the zinc finger motif (Fig. 2), although the functional significance of this domain in mammalian Lnp proteins is currently unknown.

The mammalian Lnp (mLnp1) protein features an N‐terminal myristoylation site, and ER network changes induced by overexpression of LNP proteins were significantly inhibited by the mutation of protein *N*‐myristoylation which rendered this motif nonfunctional (Moriya *et al*., 2013). Yeast Lnp1p protein, by contrast, does not feature this motif (Moriya *et al*., 2013). Arabidopsis LNP proteins similarly do not possess an N‐terminal glycine and therefore are predicted to lack a myristoylation site as shown for mLnp1 (Moriya *et al*., 2013).

The characteristic LNP amino acid motif (LNKPKH in AtLNP1 and 2) is conserved throughout the Embryophyta (land plants, i.e. flowering plants and mosses) group, although some variation in specific residues exists between species. The Plaza 4.0 online platform (Proost *et al*., 2009) was used to identify 87 genes orthologous to LNP1 and LNP2 in 49 species within the land plant lineage (from a total of 53 Embryophyta genomes present in the Plaza database). Multiple sequence alignment of these LNP proteins produced a consensus sequence motif of ALNXPK[Q/H] (Fig. S3). The existence of multiple LNP homologues within a plant species is not unique to Arabidopsis, as 30 of the 49 plant species with an identified LNP gene had two or more copies in their genome with no obvious association between gene copy number and genome divergence time. However, the existence of two subfamilies of LNP proteins, which may have, for example, evolved unique functions (paralogues) is not supported by the phylogenetic analysis of the identified LNP homologues across the Embryophyta plant group (Fig. S3).

AtLNP2 is annotated as having two possible splice variants (AtLNP2.1 and AtLNP2.2) with AtLNP2.2 containing an additional 34 residues downstream of the TMDs. However, this region is not well conserved in LNP proteins from other organisms and, moreover, publicly available RNASeq data (Araport) obtained from a range of tissues show no detectable sequence reads for this region. Hence, all subsequent analysis was performed on splice variant AtLNP2.1 (subsequently referred to as AtLNP2).

### AtLNP1 and AtLNP2 localize to different substructures within the ER network

**Figure 3:**
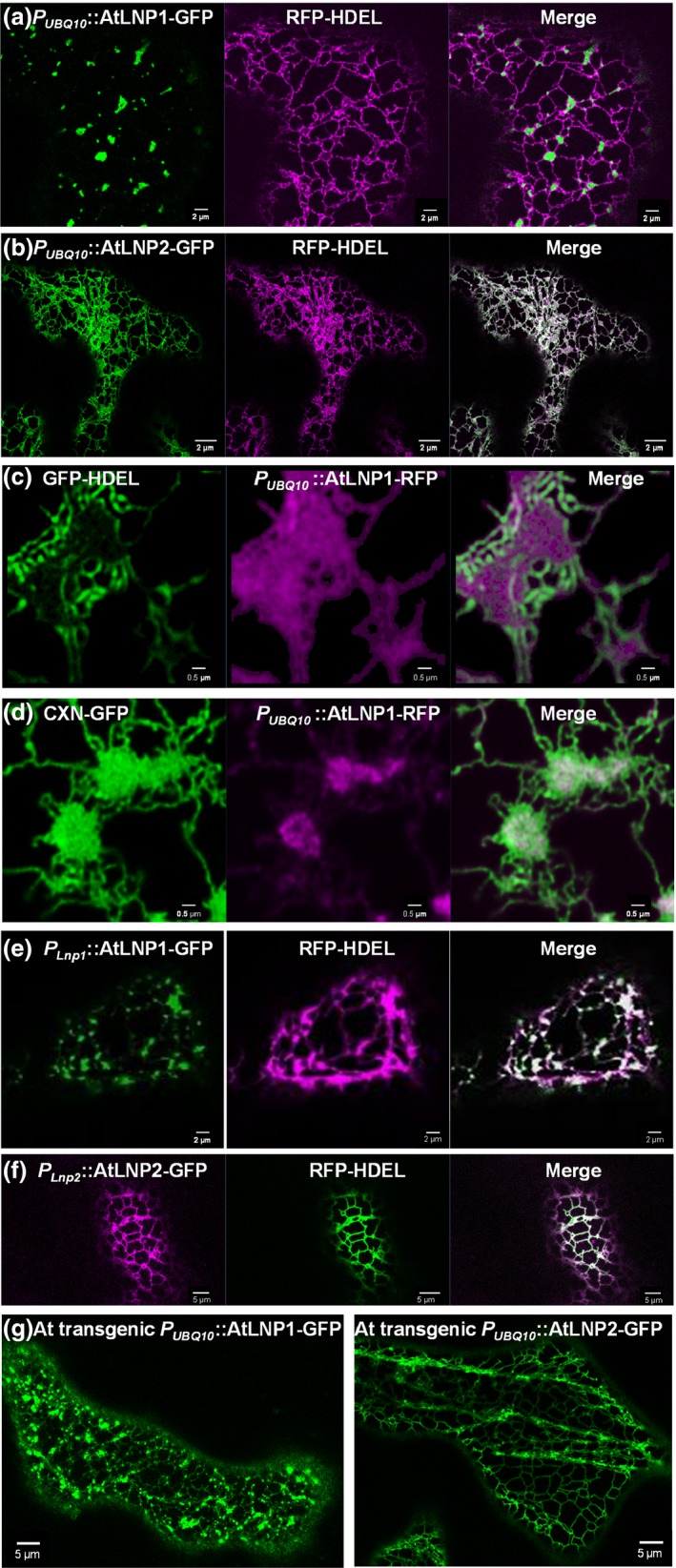
Localization of AtLNP1 and AtLNP2 within the endoplasmic reticulum (ER) network. (a, b) Transient coexpression of the ER luminal marker RFP‐HDEL with *P*_*UBQ*__*10*_::AtLNP1‐GFP (a) or *P*_*UBQ*__*10*_::AtLNP2‐GFP (b) in *Nicotiana tabacum* leaf epidermal cells. Both AtLNP1 and AtLNP2 are localized to ER cisternae and a proportion of three‐way junctions, but AtLNP2 additionally localizes to ER tubules. (c, d) High‐resolution imaging shows that AtLNP1 is more localized to the middle of the cisternae, with the lumenal marker HDEL getting pushed towards the edges (c), whereas the ER membrane marker calnexin (CXN) also labels the centre of the cisternae but expands more towards the edges than AtLNP1 (d). (e–g) Similar localization patterns are shown for *P*_*LNP*__*1*_::AtLNP1‐GFP (e) or *P*_*LNP*__*2*_::AtLNP2‐GFP (f) in *N. tabacum* and Arabidopsis lines stably expressing *P*_*UBQ*__*10*_::AtLNP1‐GFP or *P*_*UBQ*__*10*_::AtLNP2‐GFP, respectively (g).

To determine the subcellular location of AtLNP1 and AtLNP2, the full‐length protein sequences were fused at the C‐terminus to GFP under the control of the ubiquitin‐10 promoter (*P*_*UBQ10*_) (Grefen *et al*., 2010). Transient expression of both constructs in *Nicotiana tabacum* leaf epidermal cells alongside the ER lumenal marker RFP‐HDEL showed specific subcellular localization of AtLNP1 mainly to ER cisternae (Fig. 3a) as well as to a small proportion of three‐way junction regions of the ER network (which could also be small cisternae). By contrast, AtLNP2 labels the whole ER network, including cisternae (Fig. 3b).

At higher resolution, At LNP1 appears to be evenly distributed over the cisternae when coexpressed with the lumenal marker GFP‐HDEL (Fig. 3c); when coexpressed with the transmembrane region of the ER membrane marker calnexin (GFP‐CXN) AtLNP1 levels appear elevated over the cisternae compared with the tubular structure surrounding the cisternal core (Fig. 3d).

At higher resolution it can be observed that AtLNP1 localizes to the centre of cisternae whereas the lumenal marker GFP‐HDEL labels the edges of cisternae (Fig. 3c). By contrast, the transmembrane region of the ER membrane marker calnexin (GFP‐CXN) labels the whole of the cisternae and is not restricted to the centre of the membrane as is AtLNP1 (Fig. 3d).

The two different LNP localization patterns observed, where AtLNP1 is cisternae‐specific but AtLNP2 labels the whole ER network, were also found when using both native promoter constructs (2 kb upstream from gene; Fig. 3e,f) in transient expression assays and in Arabidopsis plants stably expressing AtLNP1 or AtLNP2, respectively (Fig. 3g).

### Expression of Arabidopsis LNP proteins induces cisternae in a dose‐dependent manner

**Figure 4:**
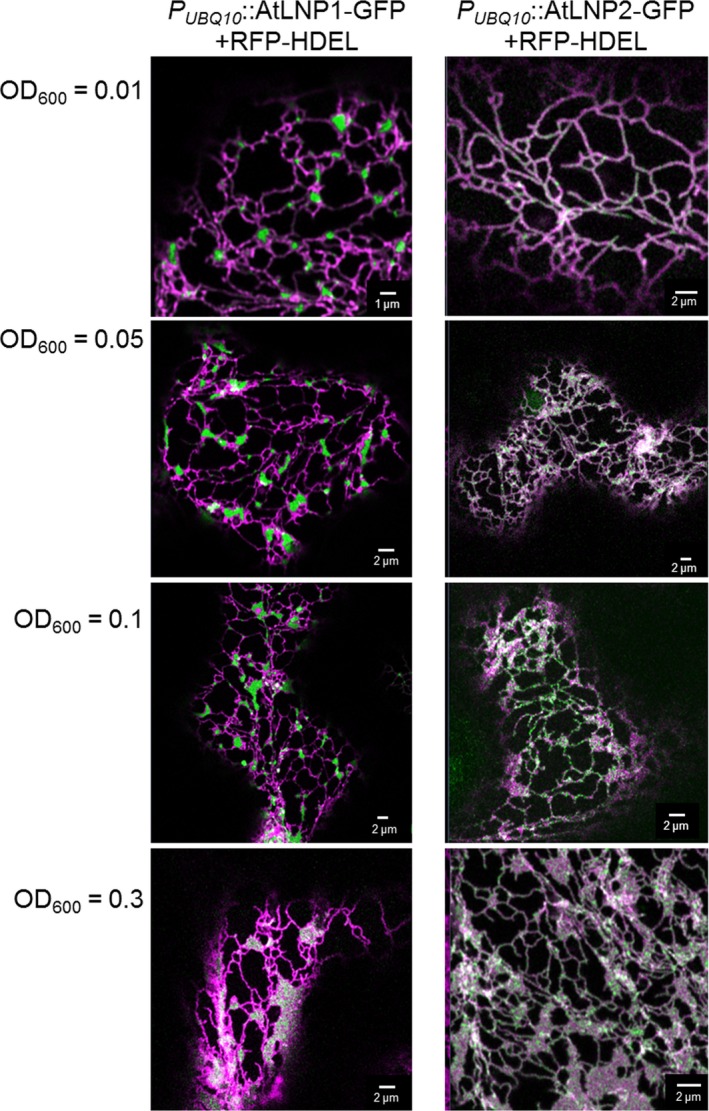
Increasing levels of protein expression affect the localization of AtLNP1 and AtLNP2 within the endoplasmic reticulum (ER) network. Transient coexpression of *Agrobacterium tumefaciens* transformed with *P*_*UBQ*__*10*_::AtLNP1‐GFP (left panels) and *P*_*UBQ*__*10*_::AtLNP2‐GFP (right panels), respectively, at increasing ODs (alongside the ER luminal marker, RFP‐HDEL at a constant OD of 0.1) in *Nicotiana tabacum* leaf epidermal cells. At higher ODs, an increasing formation of ER cisternae is observed for both constructs, and, for AtLNP1, additional labelling of ER tubules, which is absent at lower ODs.

The effect of protein expression dosage on the targeting of AtLNP1 and AtLNP2 to different ER structures was investigated by transient expression of *P*_*UBQ10*_::AtLNP1‐GFP and *P*_*UBQ10*_::AtLNP2‐GFP constructs across a range of Agrobacterium optical densities (ODs), as a proxy for differing protein expression levels within the agroinfiltrated leaf (Figs 4, S4). At higher doses of *P*_*UBQ10*_::AtLNP1‐GFP, AtLNP1‐labelled sheets became increasingly prominent with the prevalence of ER cisternae at the highest OD of the *P*_*UBQ10*_::AtLNP1‐GFP construct (Figs 4, S4a). Moreover, at higher levels of AtLNP1 expression, some AtLNP1‐labelled ER tubules were additionally observed (Figs 4, S4a).

Protein dosage effects were also seen for AtLNP2, similarly achieved through agroinfiltration of *P*_*UBQ10*_::AtLNP2‐GFP at increasing ODs. At higher construct concentrations, AtLNP2‐GFP labelling of ER three‐way junctions became increasingly prevalent, together with a marked escalation of ER cisternae formation (Figs 4, S4b).

### amiRNA *lnp1* and *lnp1lnp2* loss‐of‐function mutant shows altered ER network morphology

Given the abundance of LNP proteins at ER cisternae and their ability to induce cisternae formation when expressed at high levels *in vivo*, we hypothesize that down‐regulation of LNP could affect cisternal areas and potentially the overall appearance of the ER network. The effect on the ER network morphology in the absence of LNP proteins was therefore examined. Stable Arabidopsis homozygous lines overexpressing GFP‐HDEL and artificial microRNAs (amiRNA) designed to target either the conserved zinc finger region in both At*LNP1* and At*LNP2* (*lnp1lnp2*) or a region immediately downstream of TMD2 (*lnp1*) were generated (Fig. S1). Gene expression analysis showed expression levels of the relevant At*LNP* transcripts to be significantly reduced in these lines (Fig. S1). Loss of LNP had no noticeable effect on plant growth; unlike, for example, *rhd3* mutants which exhibit defects in plant development and are dwarfed (Zhang *et al*., 2013).

**Figure 5:**
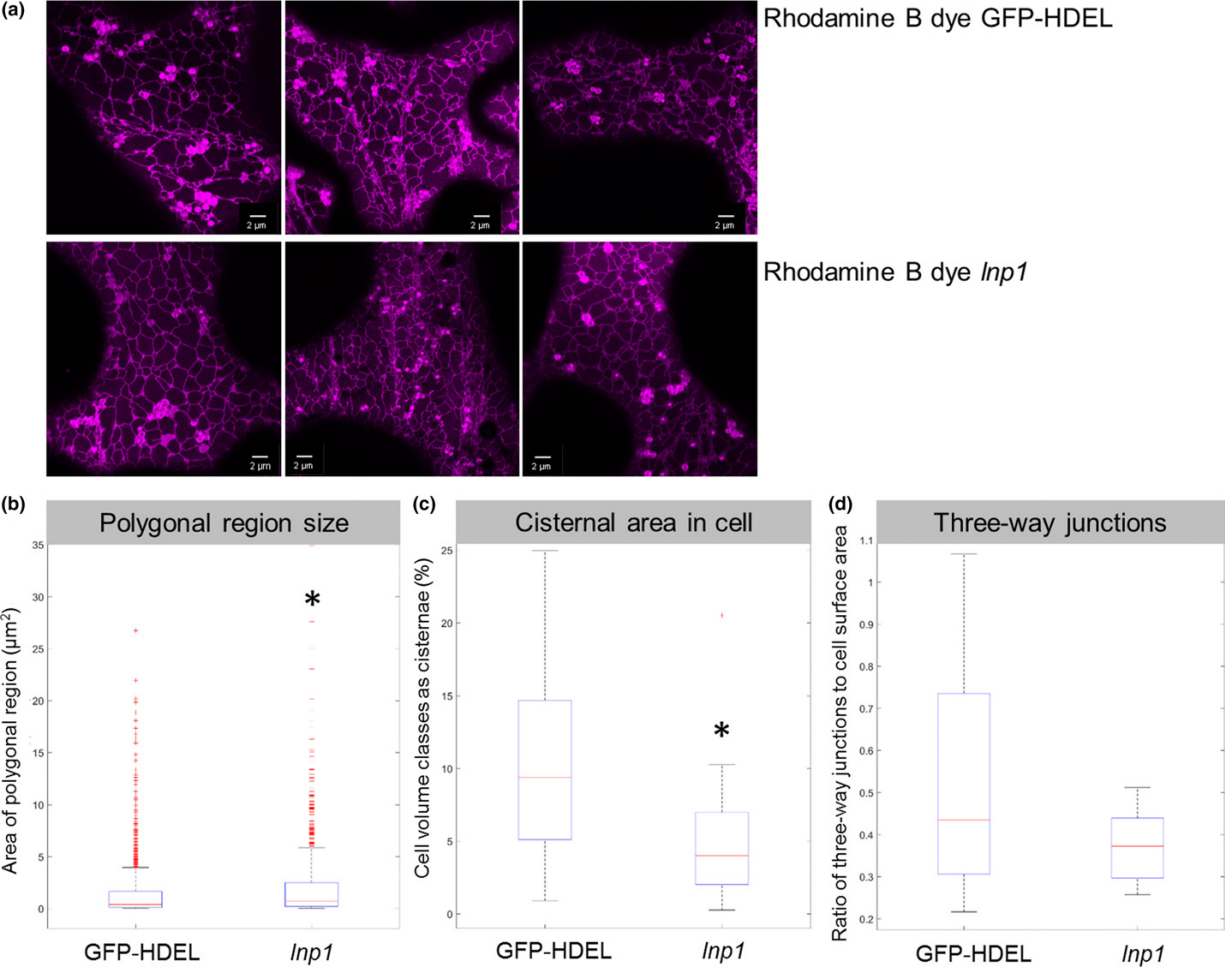
Endoplasmic reticulum (ER) network structure in Arabidopsis *lnp1* amiRNA mutants. (a) The ER network in cotyledons of GFP‐HDEL Arabidopsis plants and a stable homozygous *lnp1* amiRNA line was visualized with the lipid dye Rhodamine B hexyl ester. This dye labels the ER network but also mitochondria. Quantifications were carried out for: (b) the areas of the polygonal regions; (c) the percentage of cisternal areas in the cell volume; and (d) the ratio of three‐way junctions to cell surface area. The red horizontal mark shows the data median, and bottom and top edges of the box mark the 25^th^ and 75^th^ percentiles, respectively. The whiskers on the dashed lines outline indicate the most distant data points that are not considered outliers. Outliers are plotted individually with ‘+’ or ‘−’ symbols, respectively. *, statistical significance.

The ER network in the stable homozygous *lnp1* amiRNA line was compared with Col‐0 plants transformed with GFP‐HDEL (Fig. 5). Cells from both lines were stained with the lipid dye Rhodamine B hexyl ester (Fig. 5a), which stains the ER but also mitochondria. Network analysis (Fig. 5b–d) revealed that *lnp1* mutants have a trend towards larger polygonal regions (mean size: GFP‐HDEL 1.45 μm^2^, *lnp1* 2.05 μm^2^), reduced cisternae area per total cell area (mean percentage: GFP‐HDEL 10.69%, *lnp1* 5.62%) and reduced numbers of three‐way junctions per area (mean ratio: GFP‐HDEL 0.53, *lnp1* 0.37). *t*‐tests corrected for unequal variances were used to assess whether the ER features differed significantly between the knockdown lines and the wild‐type. This analysis showed that polygonal region area is increased and cisternal areas are decreased in *lnp1* mutants in a statistically significant manner (Fig. 5b) compared with Col‐0 plants but the number of three‐way junctions in *lnp1* mutants are not statistically significantly different from wild‐type cells at a 0.05 confidence level (Fig. 5c,d).

**Figure 6:**
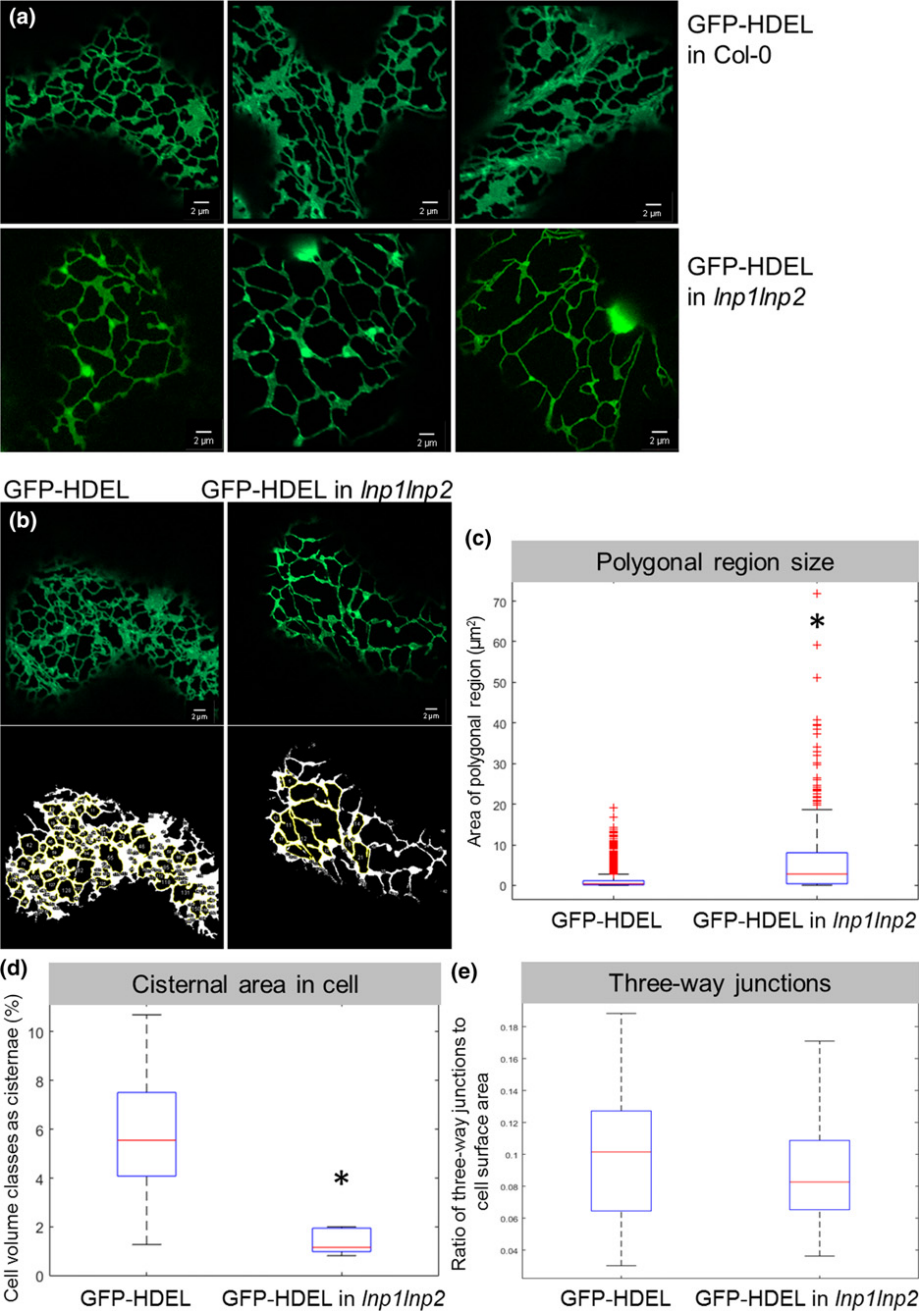
Endoplasmic reticulum (ER) network structure in Arabidopsis *lnp1lnp2* amiRNA mutants. (a) The ER network in cotyledons of Col‐0 and stable homozygous *lnp1lnp2* amiRNA lines were visualized by transformation with the ER luminal marker GFP‐HDEL. Representative images for GFP‐HDEL in the wild‐type Col‐0 and in the *lnp1lnp2* amiRNA plants are shown. (b) The areas of the polygonal regions in the ER network are outlined by GFP‐HDEL in the wild‐type Col‐0 and in the *lnp1lnp2* amiRNA plants. Polygonal areas were quantified using ImageJ's ‘Analyse particles’. The original confocal images are shown together with images post‐processing. The yellow outlines show polygonal regions with their quantifications in the box plot below. (c) As the data are not normal, the Wilcoxon rank sum test was applied. Data are shown in a box plot with a *P*‐value < 2.8930e^−44^ for three biological replicates, each with at least 12 technical replicates. In addition, the percentage of cisternal areas in the cell volume (d) as well as the ratio of three‐way junctions to cell surface area (e) were quantified. The red horizontal mark shows the data median, and bottom and top edges of the box mark the 25^th^ and 75^th^ percentiles, respectively. The whiskers on the dashed line outline indicate the most distant data points that are not considered outliers. Outliers are plotted individually with ‘+’ symbols. *, statistical significance.

Comparison of the ER network structure in cotyledons from Col‐0 and the *lnp1lnp2* knockdown line (Fig. 6), both stably transformed with GFP‐HDEL, revealed visible ER morphological differences (Fig. 6a), whereby the polygons defined by the ER tubules appeared enlarged in *lnp1lnp2*. Subsequent quantitative *in silico* image analysis (Fig. 6b) using ImageJ's ‘Analyse particles’ showed that the average ER polygonal area in *lnp1lnp2* leaves was indeed significantly greater than in the corresponding GFP‐HDEL controls (mean size: GFP‐HDEL 1.23 μm^2^, *lnp1lnp2* 6.47 μm^2^) (Fig. 6c). In addition, this analysis revealed that the ER polygonal areas are less uniform and have a greater range of sizes than in the wild‐type control (Fig. 6b,c). Analysis of the percentage of the overall cell volume classes as cisternae revealed a statistically significant reduction of cisternal areas (Fig. 6d) (mean percentage: GFP‐HDEL 5.56%, *lnp1lnp2* 1.39%) in the *lnp1lnp2* mutant and a nonsignificant trend for a reduction of three‐way junctions (Fig. 6e) in the double mutant (mean ratio: GFP‐HDEL 0.1, *lnp1lnp2* 0.09%). We hypothesize that the observed alterations in ER structure and the more irregular polygonal areas in the Arabidopsis *lnp1* and *lnp1lnp2* lines here may instead be a result of the loss of cisternae, which might have an effect on network stability rather than the nonstatistically significant reduction in three‐way junctions.

### Interaction between LNP and reticulon proteins

The ER morphology phenotypes observed upon overexpression or down‐regulation of AtLNP1 and/or AtLNP2 strongly suggest that LNP proteins are involved in the formation and/or stabilization of three‐way junctions and cisternae in the ER network. Another class of proteins known to be required for the formation of a dynamic ER tubular network in plants are the reticulon (RTN) proteins, which induce and/or stabilize membrane curvature and are capable of constricting tubules whilst suppressing cisternae formation (Sparkes *et al*., 2010). Hence, we explored the potential interplay between RTN and LNP proteins.

**Figure 7:**
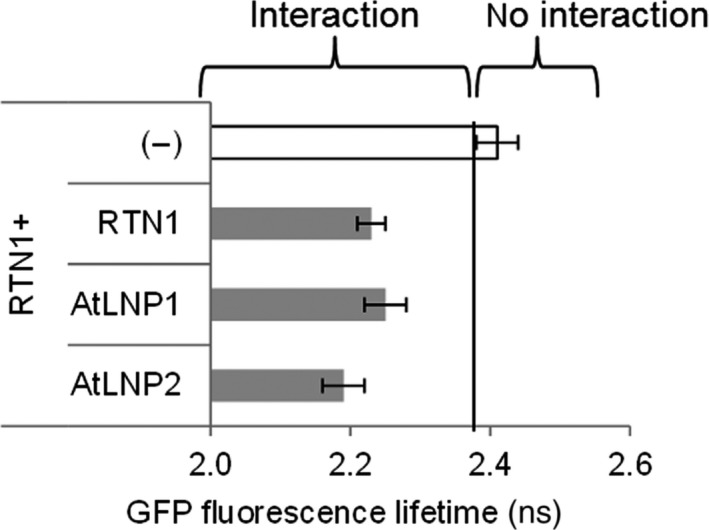
AtLNP1 and AtLNP2 form protein–protein interactions with RTN1. Combinations of donor (GFP‐RTN1) and acceptor constructs (RFP‐RTN1, AtLNP1‐RFP or AtLNP2‐RFP) were coinfiltrated into *Nicotiana tabacum* leaf epidermal cells, and protein–protein interactions were assessed by Förster resonance energy transfer by fluorescence lifetime imaging microscopy analysis. For each measurement, a region of low‐mobility endoplasmic reticulum continuous with the nuclear envelope was selected, and the fluorescence lifetime of the donor fluorophore was measured. Bar graphs depict the mean fluorescence lifetime (ns) ± SD. For each combination, at least two biological samples with a minimum of three technical replicates were used for the statistical analysis. The fluorescence lifetime of the donor construct (GFP‐RTN1) in the absence of an acceptor was used as a negative control (white bar). As RTN1 is known to form homo‐oligomers, the fluorescence lifetime of GFP‐RTN1 in the presence of the RFP‐RTN1 acceptor was used as a positive control. Lifetimes significantly lower than those of GFP‐RTN1 alone (left side of the black line) indicate protein–protein interactions.

Initially, the formation of protein–protein interactions between RTN1 and both LNP proteins was tested using FRET‐FLIM analysis *in vivo* (Figs 7, S5). RTN1 was selected as an exemplar of all RTN proteins as it has high sequence homology to other family members and is known to be expressed in all tissues throughout development (Arabidopsis eFP Browser; Winter *et al*., 2007).

Time‐resolved fluorescence spectroscopy in imaging biological systems allows for the implementation of FLIM. FRET‐FLIM measures the reduction in the excited‐state lifetime of GFP (donor) fluorescence in the presence of an acceptor fluorophore (e.g. mRFP) that is independent of the problems associated with steady‐state intensity measurements. Reduction in GFP lifetime is an indication that the two proteins are within a distance of 1–10 nm, thus indicating a direct physical interaction between the two protein fusions (Sparkes *et al*., 2010; Schoberer & Botchway, 2014). It was previously shown that a reduction of as little as 200 ps in the excited‐state lifetime of the GFP‐labelled protein represents quenching through a protein–protein interaction (Stubbs *et al*., 2005).

Donor and acceptor constructs were coinfiltrated into *N. tabacum* leaf epidermal cells and FRET‐FLIM analysis performed after 48 h to assess protein–protein interactions. For both AtLNP1 and AtLNP2, a significant reduction of 0.2 ns in the lifetime of the donor (GFP‐RTN1) fluorescence in the presence of the acceptor fluorophore (RFP‐RTN1 as a positive control and AtLNP1‐RFP, AtLNP2‐RFP) was observed, in comparison to expression of the donor alone (Figs 7, S5). These data suggest that AtLNP1 and AtLNP2 are both capable of physically interacting with RTN proteins *in vivo*.

**Figure 8:**
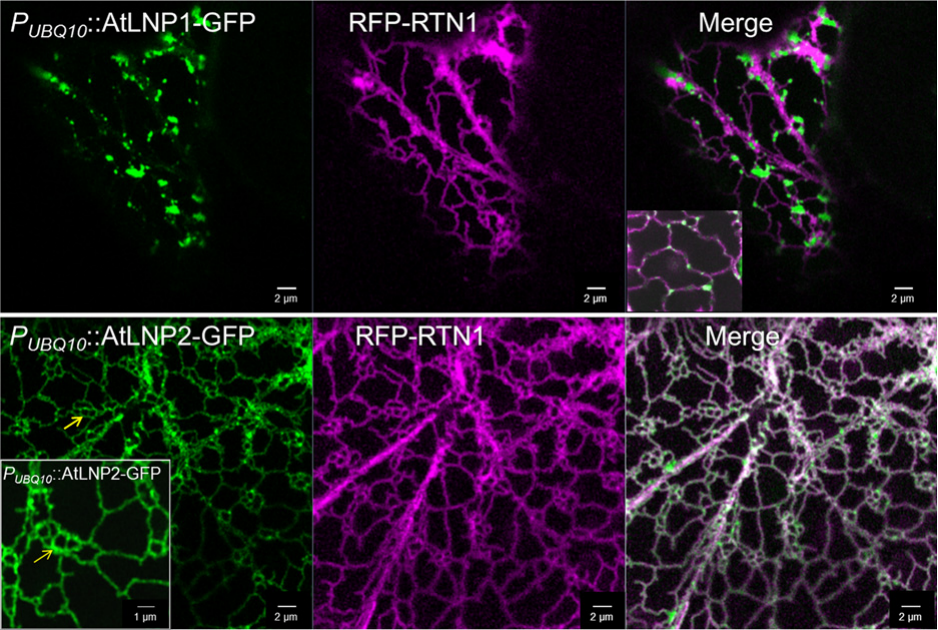
Overexpression of RTN1 influences the localization of both AtLNP1 and AtLNP2. AtLNP1‐GFP (*P*_*UBQ*__*10*_::AtLNP1‐GFP, OD = 0.3) and AtLNP2‐GFP (*P*_*UBQ*__*10*_::AtLNP2‐GFP, OD = 0.3) were agroinfiltrated into *Nicotiana tabacum* leaf epidermal cells alongside RFP‐RTN1 (35S::RFP‐RTN1, OD = 0.1). Transient coexpression of AtLNP1 together with RTN1 results in the loss of large AtLNP1‐labelled cisternae usually seen at this optical density. Coexpression of RTN1 and AtLNP2, which is less sheet‐specific and also localizes to endoplasmic reticulum (ER) tubules, results in the formation of ER nodules (yellow arrow). Insets are magnifications of portions of the images.

As FRET‐FLIM analysis demonstrated that LNP and RTN1 proteins interact *in vivo*, the existence of a possible relationship between the two proteins which influences ER morphogenesis was evaluated *in planta*. AtLNP1 and AtLNP2 constructs were expressed using an OD_600_ of 0.3 that usually results in the formation of enlarged cisternae (Fig. 4), RTN1 was infiltrated at a standard OD_600_ of 0.1 known to result in ER tubule restriction (Sparkes *et al*., 2010). Transient coexpression of AtLNP1‐GFP with RFP‐RTN1 (Fig. 8) appeared to suppress the proliferation of AtLNP1‐labelled cisternae previously observed from infiltration of AtLNP1‐GFP alone (Fig. 8, compare with Fig. 4). Instead AtLNP1‐GFP labelling of smaller, partially fragmented cisternae was detected. As observed when AtLNP2‐GFP was infiltrated on its own (Fig. 4), coexpression of AtLNP2‐GFP with RFP‐RTN1 resulted in the presence of both AtLNP2‐containing sheets and tubules. In addition, overexpression of AtLNP2 together with RTN1 induced the formation of nodule‐like structures in the ER tubular network (Fig. 8).

These data suggest that RTN1 is capable of counteracting the sheet‐induction upon LNP overexpression, further strengthening the case for these proteins having linked roles *in vivo*, such as maintaining the balance between tubules and cisternae in the network.

### Dynamics of LNP‐labelled cisternae

Yeast and mammalian Lnp proteins preferentially localize to the three‐way junctions of the ER network and it has been suggested that in these organisms Lnp proteins are involved in stabilizing these intersections (Chen *et al*., 2012, 2015; Wang *et al*., 2016). Although our results showed that AtLNP1 preferentially localizes to ER cisternae rather than junctions, we wanted to determine if AtLNP1 still has a comparable function in stabilizing the overall ER network.

The movement and remodelling dynamics of AtLNP1‐labelled ER structures was investigated through time‐lapse image processing. In general, several different types of motion were observed for Arabidopsis LNP proteins that can be classified in similar categories, as previously described for the mammalian Lnp1, despite the proteins most likely labelling different structures of the network (Chen *et al*., 2015). These movements consist of stationary Brownian‐like behaviour; directed movement of labelled regions along a tubule; merging of two adjacent puncta into a single punctum, and absorption and separation (mainly for cisternae) of discrete puncta into two independent puncta (Fig. S6).

Further analysis of videos compiled from the time‐lapse images (total of 30 images) revealed that out of the 90 AtLNP1‐labelled puncta (junctions or smaller cisternae) analysed in 20 videos, 59 (66%) moved from their original position. The remaining 31 (34%) puncta stayed fixed in their location and did not move. Moreover, within the duration of the time‐lapse experiment, 16 of these 90 AtLNP1‐labelled puncta also fused together, whilst seven puncta were absorbed into ER sheets. This appears to differ from the mammalian system cells where a significant majority (74%) of stable junctions are labelled with mLnp1 and < 6% of unstable junctions actually acquire mLnp1 (Chen *et al*., 2015).

**Figure 9:**
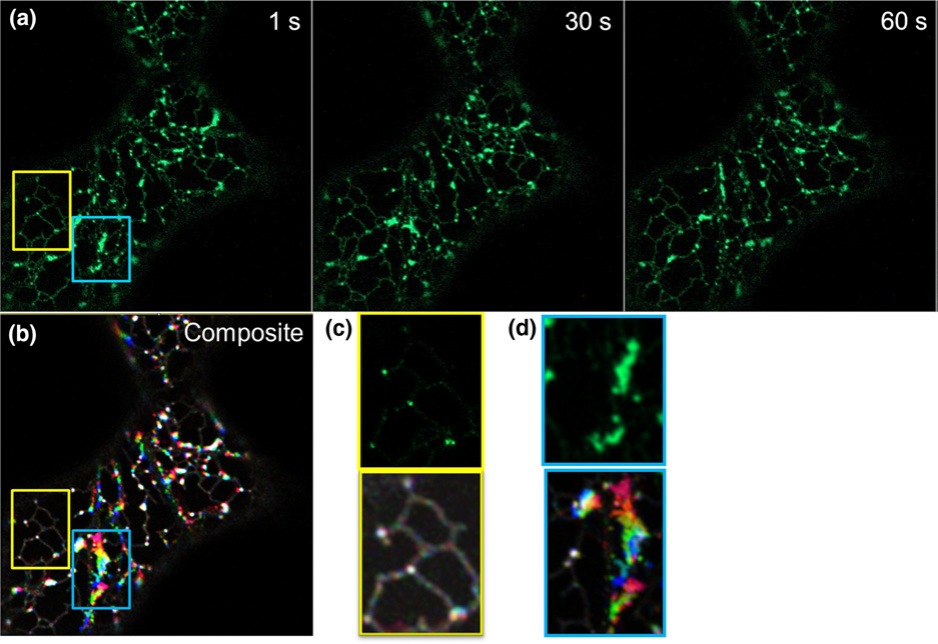
Persistency mapping of AtLNP1‐labelled endoplasmic reticulum (ER) cisternae and three‐way junctions. (a) *P*_*UBQ*__*10*_::AtLNP1‐GFP was transiently expressed in *Nicotiana tabacum* leaf epidermal cells. Videos of ER cisternae labelled with *P*_*UBQ*__*10*_::AtLNP1‐GFP were taken over 60 s. Static images of the AtLNP1‐GFP‐labelled ER network are shown at 1, 30 and 60 s. (b) Composite image of the three time points shown in (a) following temporal colour coding of the video (performed in the ImageJ ‘temporal color‐code’ plugin). White areas indicate points of persistency occurring for the duration of the time imaged, and coloured areas indicate movement, with areas in red showing the highest mobility. (c) Magnification of a stable region of the ER (framed in yellow in b). Confocal image of AtLNP1‐GFP‐labelled three‐way junctions (top) and the corresponding persistency map (bottom). Within this relatively stable area of the ER network, both AtLNP1‐labelled junctions and junctions with no detectable AtLNP1 expression remain persistent. (d) Magnification of a highly motile region of the ER undergoing rapid remodelling (framed in blue in b). Confocal image of AtLNP1‐GFP‐labelled three‐way junctions and cisternae (top) and the corresponding persistency map (bottom).

To test if the presence of AtLNP1 actively stabilizes the ER network or if AtLNP1‐labelled junctions and cisternae follow the overall movement of the network as a whole, the dynamics of AtLNP1‐labelled junctions and cisternae were analysed in comparison to the surrounding network (Fig. 9). Persistency mapping was performed on videos capturing the movement of AtLNP1‐GFP labelled junctions and cisternae (Fig. 9a,b). Persistency was assessed using spatiotemporal projections of AtLNP1‐GFP labelled cells over 60 s with analysis performed on both relatively stable (Fig. 9c) and more dynamic (Fig. 9d) regions of the cortical ER network with cytoplasmic streaming. In the more stable areas of the ER where remodelling was minimal, AtLNP1‐labelled ER elements similarly exhibited limited mobility and thus had high persistency (Fig. 9b,c). It should also be noted that stable three‐way junctions and polygons were frequently observed not to have acquired AtLNP1. In areas of high motility, AtLNP1‐labelled areas also show high motility and low persistency (Fig. 9b,d).

**Figure 10:**
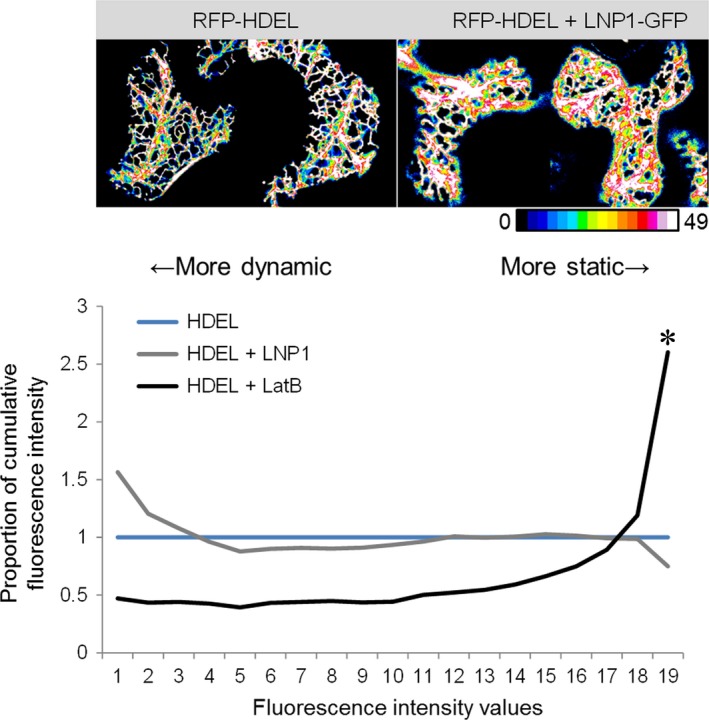
Endoplasmic reticulum (ER) network dynamics with and without AtLNP1 protein. ER network persistency was analysed in *Nicotiana tabacum* leaf epidermal cells for RFP‐HDEL alone (blue line), together with AtLNP1 (grey line), as well as after application of the drug Latrunculin B (black line) which disrupts F‐actin formation and has been shown to stop/slow the ER network. Cumulative fluorescence intensity (CFI) values of RFP‐HDEL networks are calculated using cumulative fluorescence intensity; a higher proportion of higher CFI values indicates a more static network, while a higher proportion of lower CFI values means a more dynamic network. Colour‐coded example images are shown at the top as a heat map: white shows pixels that were occupied during the entire time‐series (50 frames), and blue indicates pixels that were only occupied for a few time frames. The graph represents the CFI distribution. All conditions are expressed as a ratio compared with the RFP‐HDEL control, hence the RFP‐HDEL curve itself is always one. Data indicate that AtLNP1 expression does not affect ER network dynamics, but Latrunculin B significantly slows down ER network movement (indicated by asterisk *). *n *=* *10.

We also investigated if the expression of AtLNP1 has an influence on the overall ER network dynamics. For this, videos were acquired (Fig. S7) and the ER network persistency was analysed for RFP‐HDEL with and without AtLNP1‐GFP (Fig. 10). As a control, Latrunculin B, which disrupts F‐actin formation and has been shown to stop/slow the ER network, was used together with RFP‐HDEL. Cumulative fluorescence intensity (CFI) values of RFP‐HDEL (with and without AtLNP1 or Latrunculin B) networks were calculated (Tolmie *et al*., 2017); in this analysis a higher proportion of higher CFI values indicates a more static network, while a higher proportion of lower CFI values means a more dynamic network. Only Latrunculin B treatment, but not coexpression with AtLNP1, resulted in a significant increase of network persistency, indicating that AtLNP1 does not change network dynamics (Fig. 10).

Taken together, these data indicate that, unlike in mammalian systems, ER three‐way junctions and network stability are not dependent on the presence of AtLNP1, but that the motility of AtLNP1‐labelled junctions and cisternae follows the motility or persistency of the surrounding ER network architecture structure.

## Discussion

Here we have reported the identification and characterization of two plant homologues (AtLNP1 and AtLNP2) of the mammalian and yeast LNP proteins.

### Subcellular location of Arabidopsis LNP proteins

AtLNP1 localizes mainly to cisternae and a small proportion of three‐way junctions in the ER network. This is in contrast to the subcellular localization described for yeast and mammalian Lnp proteins which is rather specific to three‐way junctions; yeast and mammalian Lnp proteins label *c*. 50% of three‐way junctions, resulting in their stabilization (Chen *et al*., 2012; Shemesh *et al*., 2014; Wang *et al*., 2016). Both AtLNP1 and its animal counterpart can also label ER tubules at higher protein expression levels (Shemesh *et al*., 2014; Wang *et al*., 2016). By contrast, AtLNP2 expression was observed throughout the ER network, in both cisternae and tubules, suggesting that, despite sharing 67% protein sequence homology with AtLNP1, they are most likely not complete functional homologues. Single knockdown *lnp1* mutants display a less visible morphological phenotype than the double *lnp1lnp2* mutant (Figs 5, 6), but nonetheless the increase in polygonal area size and the decrease in the amount of cisternal areas are statistically significant (Fig. 5). Polygonal areas are increasing 1.4‐fold in *lnp1* but over fivefold in *lnp1lnp2* compared with Col‐0, and cisternal areas are reduced by 50% in *lnp1* but by 75% in *lnp1lnp2* compared with Col‐0. As the additional knockdown in AtLNP2 seems to enhance the ER network phenotype, this could indicate some degree of functional redundancy. Furthermore, to date, no second LNP protein with an ER tubular localization has been described in yeast and mammalian systems that would correspond to AtLNP2, raising the question about function and redundancy of the second Arabidopsis LNP protein. Phylogenetic analysis of LNP orthologues in other land plant species also failed to reveal the existence of two (or more) obvious subgroups of LNP proteins which may have, for example, developed distinct functionality. *Homo sapiens*, by contrast, code for at least five Lnp isoforms, which may potentially have different spatiotemporal expression patterns and/or functional specialization.

### LNP function in the ER network

Overexpression of Arabidopsis LNP proteins results in increased cisternae formation in a dose‐dependent manner (Fig. 4). By contrast, in mammalian cells it has previously been demonstrated that increasing amounts of mLnp1 protein result initially in ER cisternae induction, followed by the generation of thicker ER tubules at higher mLnp1 expression levels (Wang *et al*., 2016). In COS cells (Shemesh *et al*., 2014) mLnp1 localizes to three‐way junctions at low expression levels as well as to some punctae on tubules. Higher mLnp1 expression levels result in clustered localization to densely branched tubules, also resulting in increased numbers of three‐way junctions; at very high expression levels, Lnp localizes to longer, unbranched tubules concomitant with a decrease in three‐way junctions. No such decrease in three‐way junction abundance or thicker ER tubules were observed upon increased LNP expression in either tobacco or Arabidopsis (Figs 3, 4), although this may be a result of the limitations of achievable maximal protein expression levels in the transient expression system as very high doses of agrobacterium frequently result in leaf necrosis.

Analysis of both loss‐ and gain‐of‐function mutants in the two identified AtLNP proteins revealed further potential functional differences between plant and mammalian/yeast LNP proteins. Knockdown of AtLNP1 alone has a significant increase in polygonal region areas and a significant reduction in cisternae (Fig. 5). A *lnp1lnp2 *×* *GFP‐HDEL knockdown line exhibited a significant reduction in cisternal area (Fig. 6d) and a significantly increased mean ER polygonal area in comparison to the GFP‐HDEL control (Fig. 6c). The polygonal areas of the mutant also spanned a greater size distribution than those of the wild‐type control (Fig. 6c), ultimately resulting in a less structured, ‘looser’ ER network in the mutant. A trend towards fewer three‐way junctions was observed in both the *lnp1* mutant (Fig. 5d) and the *lnp1lnp2* mutant (Fig. 6e) but this was not significant in both mutants. This observed ER morphology upon depletion of AtLNP again contrasts with that described in similar studies in yeast and mammalian systems. Mutations in the zinc finger motif of yeast Lnp1p led to a reduction in polygon size and thus resulted in a densely reticulated network (Chen *et al*., 2012), and, similarly, the loss of mLnp1 gave rise to a more compact, sheet‐like ER morphology (Chen *et al*., 2015). Lnp mutant forms expressed in U2OS cells lacking Lnp show sheet generation with a reduction in tubules and junctions (Wang *et al*., 2016). Moreover, addition of cytoplasmic fragments of *Xenopus* Lnp acting as a dominant‐negative mutant to a *Xenopus* network formation resulted in the replacement of three‐way junctions by small cisternae, as well as an overall reduction in three‐way junctions (Wang *et al*., 2016). These results recently led Wang *et al*. (2016) to conclude that mammalian mLnp1 is not essential for ER tubule and junction formation but instead affects three‐way junction abundance. In the plant system we did not observe an increase in junctions (as described for yeast), or cisternae (as described in yeast and mammalian cells), but rather the opposite.

We hypothesize that the striking changes in polygonal structure and areas reported here for the Arabidopsis *lnp1* and *lnp1lnp2* knockdown lines are a result of the loss of cisternal areas rather than of the nonsignificant reduction in nascent three‐way junctions. A possible explanation for the ER morphology observed in the mutants is that depletion of AtLNP1 (and AtLNP2) results in a decrease in cisternae and/or reduction in cisternal stability, which might result in a change in the biophysical properties of the ER, leading to a less stable and structured network. This is also in agreement with the formation of enlarged cisternal structures upon overexpression of Arabidopsis LNP proteins, as described earlier (Fig. 4).

Several functions for LNP proteins have been suggested in the various systems studied and these are rather diverse. In *Caenorhabditis elegans*, mutations in lnp‐1 have been linked to neuronal defects similar to those in atlastin or the Yop1p homologue REEP1 mutants (Ghila & Gomez, 2008).

A function for mLnp1 in the stabilization of three‐way junctions has been discussed (Chen *et al*., 2015). Through immunolabelling of endogenous mLnp1, Chen *et al*. (2015) showed that the protein is only detectable in about half of the three‐way junctions in the mammalian network. The group also reported that junctions with mLnp1 are less mobile than junctions without mLnp1 and are less likely to show junction loss through ring closure (Chen *et al*., 2015). A fraction of newly formed junctions go on to acquire mLnp1 protein. Newly formed junctions that do not acquire mLnp1 have a high probability of loss through ring closure and are relatively mobile, whereas, conversely, those that do acquire mLnp1 have a greatly reduced probability of loss and are less mobile. This is especially prominent in newly formed junctions, indicating that mLnp1 stabilizes newly formed three‐way junctions but is not required to be continually present on the junction thereafter (Chen *et al*., 2015). In the absence of mLnp1, new junctions are still being formed but are less likely to persist, resulting in more sheet‐like structures (Chen *et al*., 2015). For the plant system we could not find any indication that AtLNP1 or 2 stabilizes three‐way junctions. The network dynamics is instead dependent on the surrounding network rather than on the presence or absence of LNP proteins (Fig. 9).

For mammalian cells it was also suggested that mLnp1 is not necessary for the generation or maintenance of the ER network as, in the absence of mLnp1, there is still a reticular network, even if most three‐way junctions are converted into larger cisternae (Wang *et al*., 2016). Instead, mLnp1 proteins are proposed to move into three‐way junctions with the overexpression of mLnp1 resulting in the expansion of cisternae, but atlastin proteins are suggested to be responsible for the initial formation of native junctions (Wang *et al*., 2016).

Interestingly, a theoretical model predicts mLnp1 to be a so‐called S‐type protein capable of stabilizing curvature and favouring negative curvature, which plays an important role in generating and stabilizing three‐way junctions (Shemesh *et al*., 2014). This contradicts the hypothesis of Chen *et al*. (2012) that Lnp proteins are involved in abolishing three‐way junctions in yeast. The latter was suggested following the observation that the ER in lnp1p mutants is highly reticulated resulting in an increased abundance of three‐way junctions. However, Shemesh *et al*. (2014) suggest that this might well be a cisternal structure rather than a reticulated network as the two cannot be distinguished at the resolution of the images. The model of Shemesh *et al*. (2014) is also of interest to Arabidopsis LNP proteins as cisternae are often bordered by negatively curved edge lines. In Arabidopsis we find RTN proteins on the curved edges of cisternae (Sparkes *et al*., 2010) whereas AtLNP1 labels more the flat part of the central membrane rather than the edges (Fig. 3c,d). This may hint at a mechanism for how AtLNP1 and 2 induce or stabilize cisternae and work in collaboration with RTN proteins to retain the cisternae–tubule ratio.

### Interaction with reticulons

In yeast, it is suggested that Lnp1p acts in synergy with the reticulons and Yop1p. Indeed, Lnp1p has been shown to interact with Rtn1p, Yop1p and Sey1p, as a loss‐of‐function mutation in Lnp1p in a rtn1/rtn2/yop1 triple mutant results in growth and ER morphological defects (Chen *et al*., 2012). Lnp1p physically interacts with Rtn1p, indicating that they may act on converging pathways, as mutants in these genes display different ER phenotypes: loss of Lnp1p leads to the formation of densely reticulated ER (Chen *et al*., 2012), whereas the loss of Rtn1p results in nonfenestrated sheets (De Craene *et al*., 2006).

In mammalian cells it has been proposed that Lnp proteins are not required for ER network formation but instead are involved in the formation of sheets at tubule junctions (Wang *et al*., 2016). The presence of Lnp within the three‐way junctions may then prevent the migration of reticulons into the junction, thereby preventing junction expansion; in the absence of LNP, three‐way junctions could therefore expand into large sheets (Wang *et al*., 2016).

We show here that the AtLNP1 and 2 proteins are capable of interacting with reticulons (Fig. 7) and, moreover, that the induction of large cisternal regions resulting from LNP overexpression is suppressed by coexpression of reticulon proteins (Fig. 8). We therefore hypothesize that, in Arabidopsis, LNP and reticulon proteins function in concert to maintain the ratio of ER cisternae to tubules and are capable of interconverting the two morphologies.

In conclusion, we propose that Arabidopsis LNP proteins are involved in the formation and/or stabilization of ER network cisternae. They are also highly likely to functionally cooperate with other ER morphogens such as the reticulon family of proteins, with which they interact *in planta*. One hypothetical scenario could be that LNP proteins which are labelling cisternae but not the edges (Fig. 3c,d) induce the flat membranes of cisternae, whereas reticulons that localize to the edge of cisternae (Sparkes *et al*., 2010) induce curvature, thereby limiting cisternal expansion. In contrast to that described for their yeast and mammalian orthologues, Arabidopsis LNP proteins label only a small proportion of junctions which could also potentially be small cisternae, and we did not find any evidence that they stabilize ER three‐way junctions. The presence of two LNP homologues within the Arabidopsis genome that show distinct subcellular localization but which might also have some degree of functional redundancy is of interest. We are currently investigating the impact of the different localizations of AtLNP1 and 2 on the ER and resulting functionality, as well as the interactions of the AtLNP proteins with other ER‐shaping proteins.

## Author contributions

V.K. and C.H. designed the research. E.B. and L.F. cloned constructs, generated the mutant lines and performed the LNPARK amino acid comparative motif analysis. C.P. and F.T. carried out the mutant and network movement analysis. V.K. cloned constructs and carried out confocal microscopy as well as the overexpression, persistency and FRET‐FLIM analysis. All authors contributed to the writing of the article.

## Supporting information

Please note: Wiley Blackwell are not responsible for the content or functionality of any Supporting Information supplied by the authors. Any queries (other than missing material) should be directed to the *New Phytologist* Central Office.

**Fig. S1** Analysis of *LNP* transcript abundance in Arabidopsis amiRNA lines.
**Fig. S2** Microarray data in the eFP browser for At*LNP1* and At*LNP2*.
**Fig. S3** Lunapark motif analysis in Embryophyta species.
**Fig. S4** OD expression series for AtLNP1 and AtLNP2 in tobacco epidermal leaf cells (addition to Fig. 4).
**Fig. S5** Raw data for protein–protein interactions by FRET‐FLIM (addition to Fig. 7).
**Fig. S6** AtLNP1‐labelled cisternae display different dynamic behaviours.

**Fig. S7** Example movies for ER network persistency analysis in Fig. 10.
