# Supplementary material for: Arabidopsis Lunapark proteins are involved in ER cisternae formation
Source: New Phytol. 2018 May 25;219(3):990–1004. doi: 10.1111/nph.15228 (PMC6055799; doi:10.1111/nph.15228)

***New Phytologist* Supporting Information**

Article title:

Arabidopsis Lunapark proteins are involved in ER cisternae formation.

Authors:

Verena Kriechbaumer, Emily Breeze, Charlotte Pain, Frances Tolmie, Lorenzo Frigerio, Chris Hawes

Article acceptance date: 17 April 2018

The following Supporting Information is available for this article:

Figure S1: Analysis of At*LNP* transcript levels in amiRNA lines.

A) Alignment of arabidopsis At*LNP1* (At2g24330.1) and At*LNP2* (At4g31080.1) cDNA sequences with the location of the sequences targeted by amiRNA1 and amiRNA2 indicted in blue. The conserved transmembrane domains (TMD), zinc finger, and LNPARK motif (LNKPKH in arabidopsis) are also indicated to aid comparison with the protein alignment shown in Figure 1. The sequences of the At*LNP1* and At*LNP2* specific primers used in RT-PCR analysis of the amiRNA lines are underlined.

B) RT-PCR gene expression analysis of the At*LNP1* and At*LNP2* transcript levels in amiRNA1- and amiRNA2-containing arabidopsis lines relative to wild-type (Col-0). amiRNA2 reduced the expression of both At*LNP1* and At*LNP2* and these lines were subsequently referred to as *lnp1lnp2*; whereas amiRNA1 only reduced the expression of At*LNP1* and so these lines were subsequently referred to as *lnp1*. At4g34270 and At4g12590 represent two seed-specific house-keeping genes as controls.

A

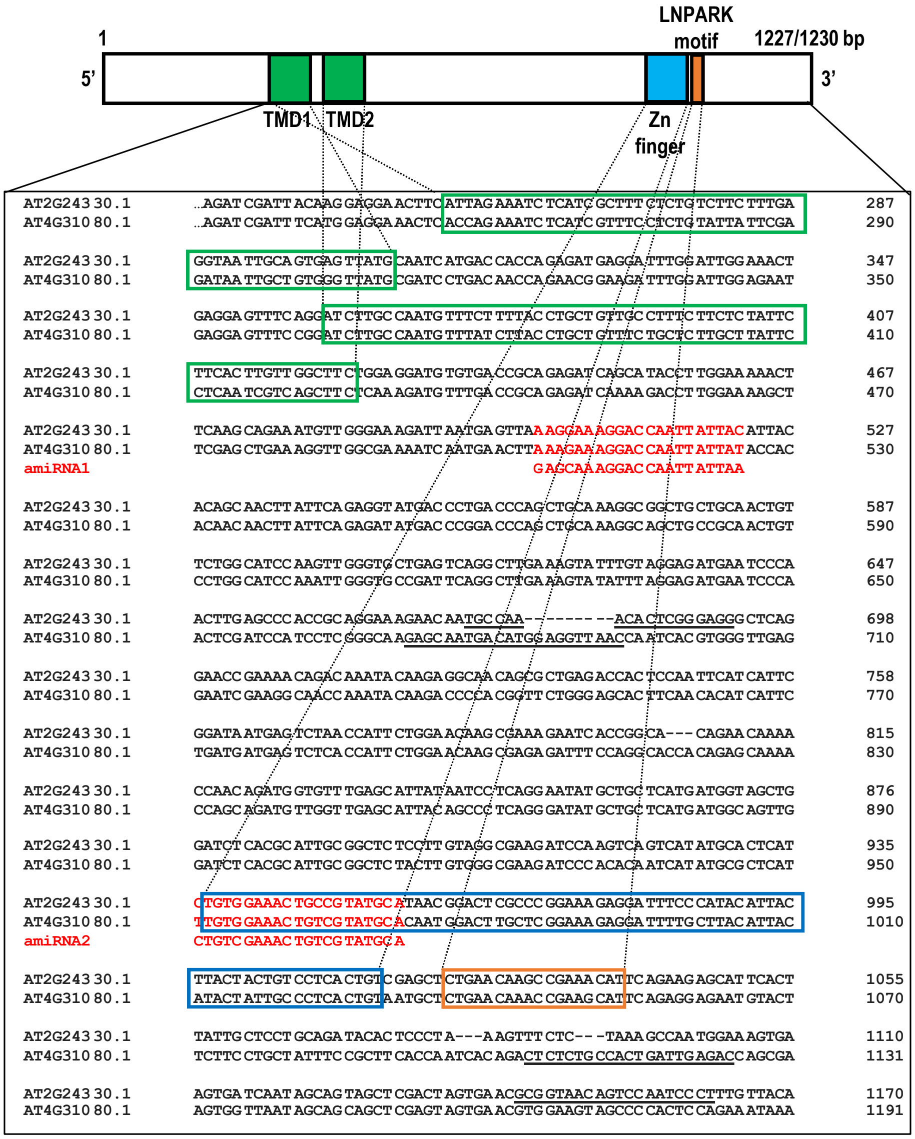

B

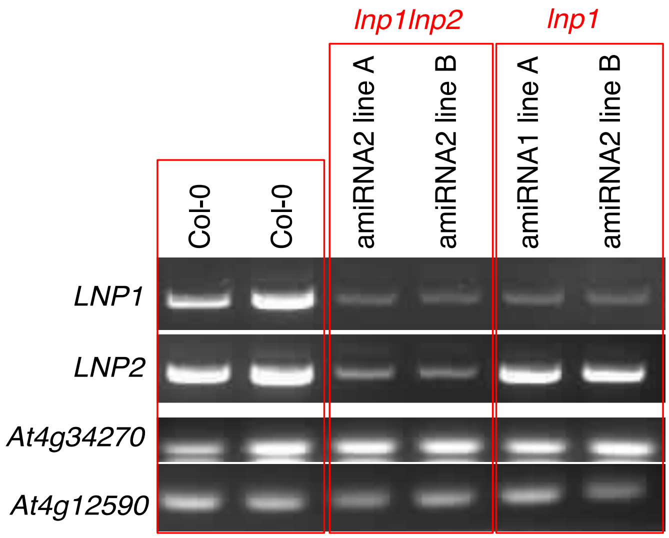
lnp1lnp2
lnp1
Col-0
Col-0
amiRNA2 line A
amiRNA2 line B
amiRNA1 line A
amiRNA2 line B
LNP1
LNP2
At4g34270
At4g12590

Figure S2: Microarray data in the eFP browser for At*LNP1* and At*LNP2*.

A) Microarray data for transcription profiles for At*LNP1* and At*LNP2* in graphic representation during plant development are shown.

B) Reverse-transcriptase cDNA gel using arabidopsis cotyledonal tissue indicates that AtLNP proteins are present in this tissue.

A

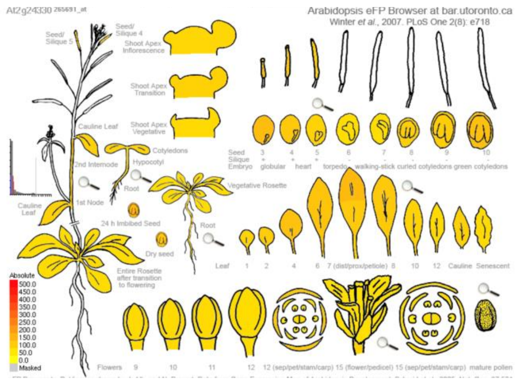

AtLNP1 (At2g24330)

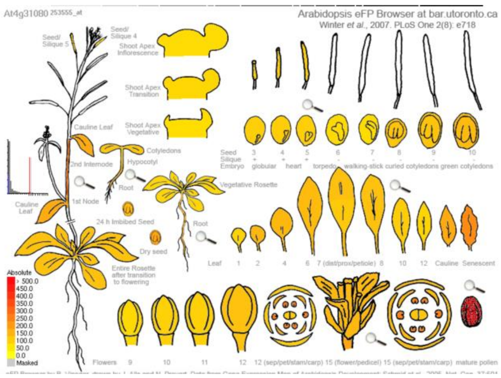

AtLNP2 (At4g31080)

B

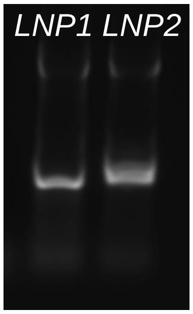

Figure S3: Lunapark motif analysis in Embryophyta species.

87 Lunapark homologs containing the InterPro Lunapark domain (IPR019273) from 49 species within the land plant lineage were identified using the PLAZA 4.0 online platform.

A) Phylogenetic tree of LNP plant orthologs. Position of InterPro Lunapark domain within the protein sequence is shown by a pink box; the LNP amino acid motif is located immediately downstream of this domain. The two arabidopsis LNP proteins are indicated by an asterisk. Note: three proteins (MDO.mRNA.g.321.9 [*Malus domestica* (MDO); apple], and Os02g58380 and Os04g44180 [*Oryza sativa* ssp. japonica; rice]) show sequence homology to other LNP proteins, notably in the TMDs and regions upstream of the zinc finger, but crucially do not contain a recognisable Cys4 type C-terminal zinc finger nor an LNP motif and so were excluded from the consensus motif analysis. The pale yellow boxes at the N-terminus of MDO.mRNA.g.321.9 represent pentatricopeptide repeats.

B) Consensus sequence logo of LNP amino acid motif from multiple sequence alignment of identified plant LNP homologs. Amino acids are coloured according to their chemical properties such that- green, polar residues; purple, neutral residues; blue, basic residues; red, acidic residues; black, hydrophobic residues.

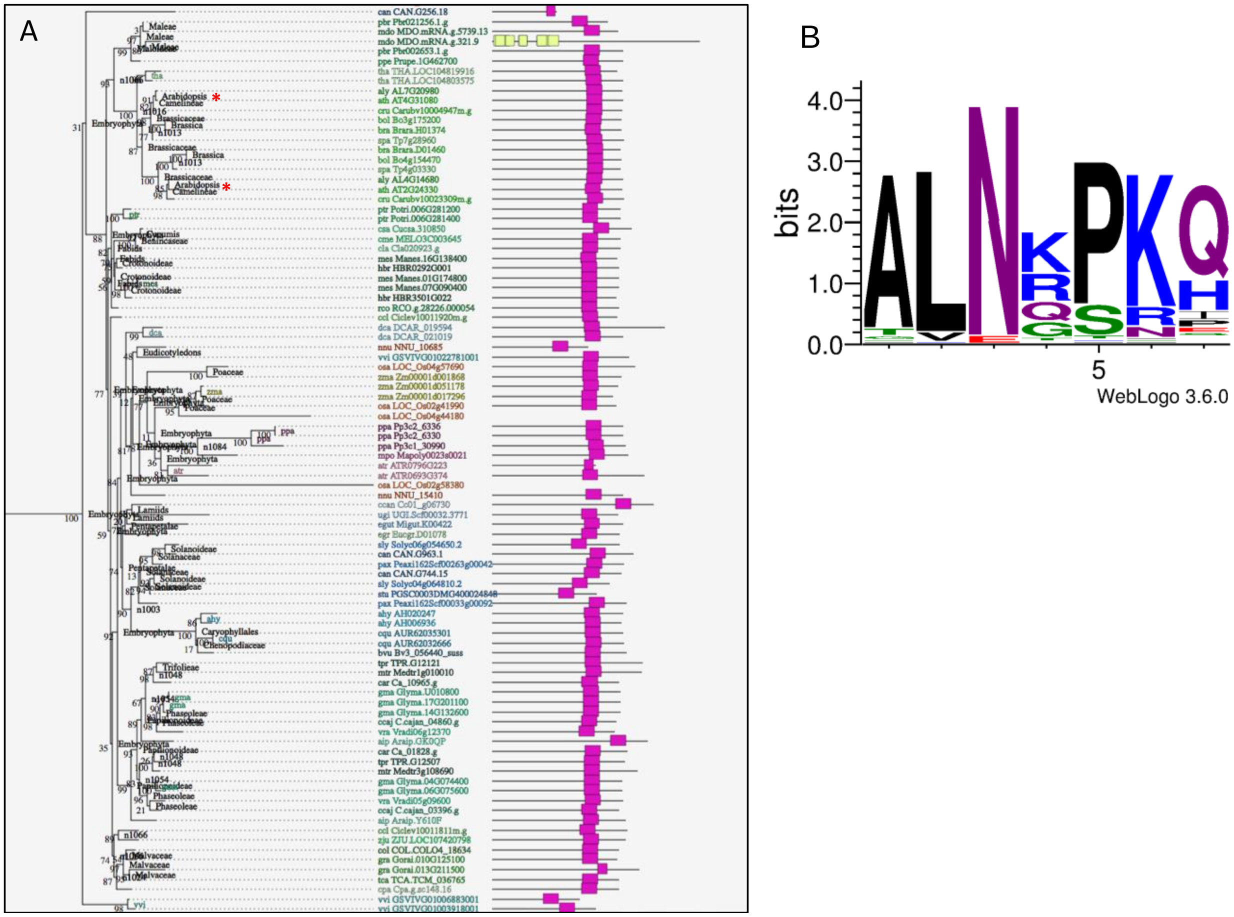

Figure S4a: OD expression series for AtLNP1 in tobacco epidermal leaf cells.
Agrobacterium tumefaciens transformed with P_{UBQ10}::AtLNP1-GFP is infiltrated in tobacco epidermal leaf cells in increasing optical densities (OD). With increasing ODs cells display an increasing number of cisternae and more labelling in the tubules. The lumenal marker RFP-HDEL is used to label the ER network. Size bars are given.

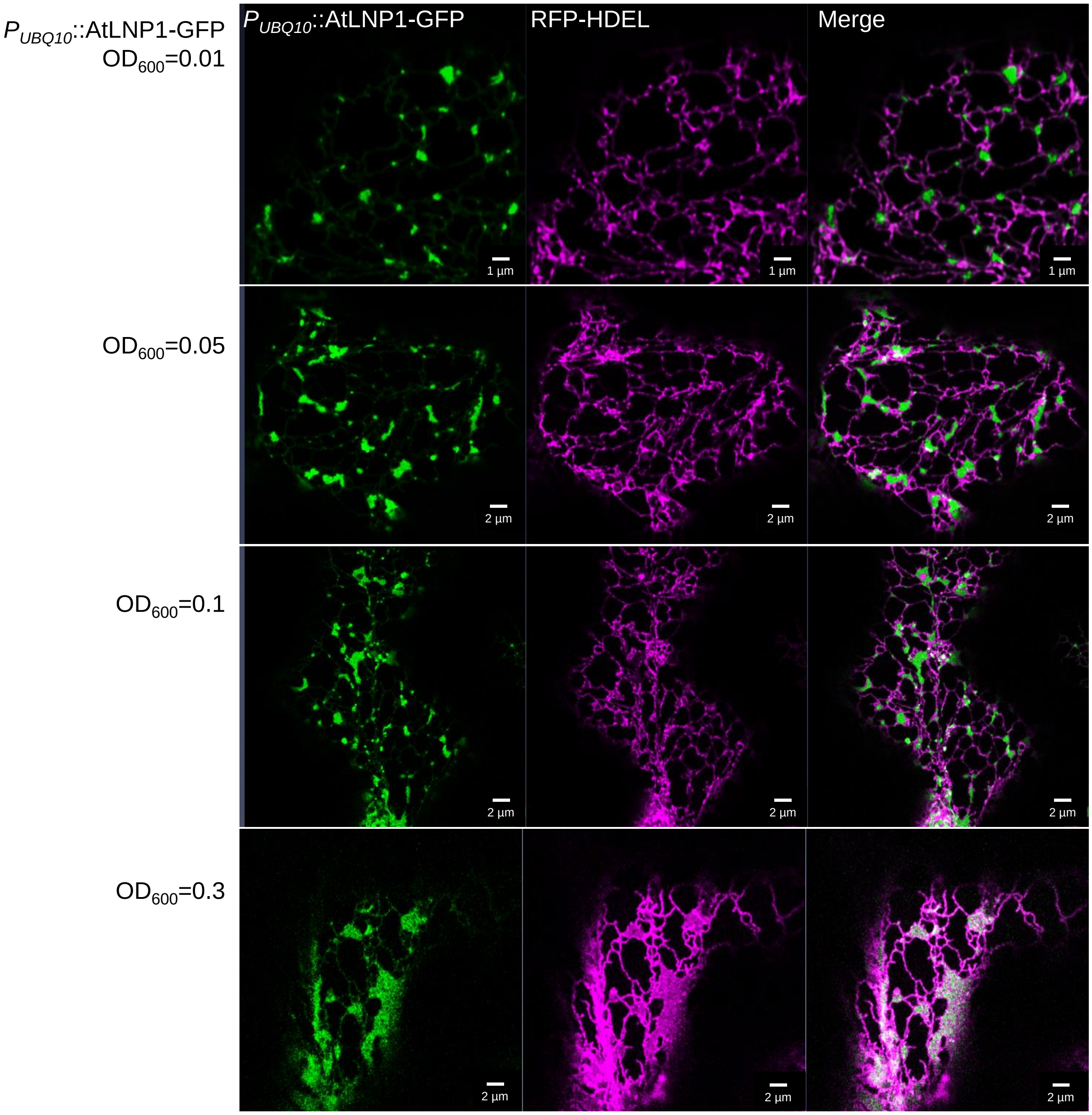

Figure S4b: OD expression series for AtLNP2 in tobacco epidermal leaf cells.
Agrobacterium tumefaciens transformed with P_{UBQ10}::AtLNP2-GFP is infiltrated in tobacco epidermal leaf cells in increasing optical densities (OD). With increasing ODs cells display an increasing number of cisternae. The lumenal marker RFP-HDEL is used to label the ER network. Size bars are given.

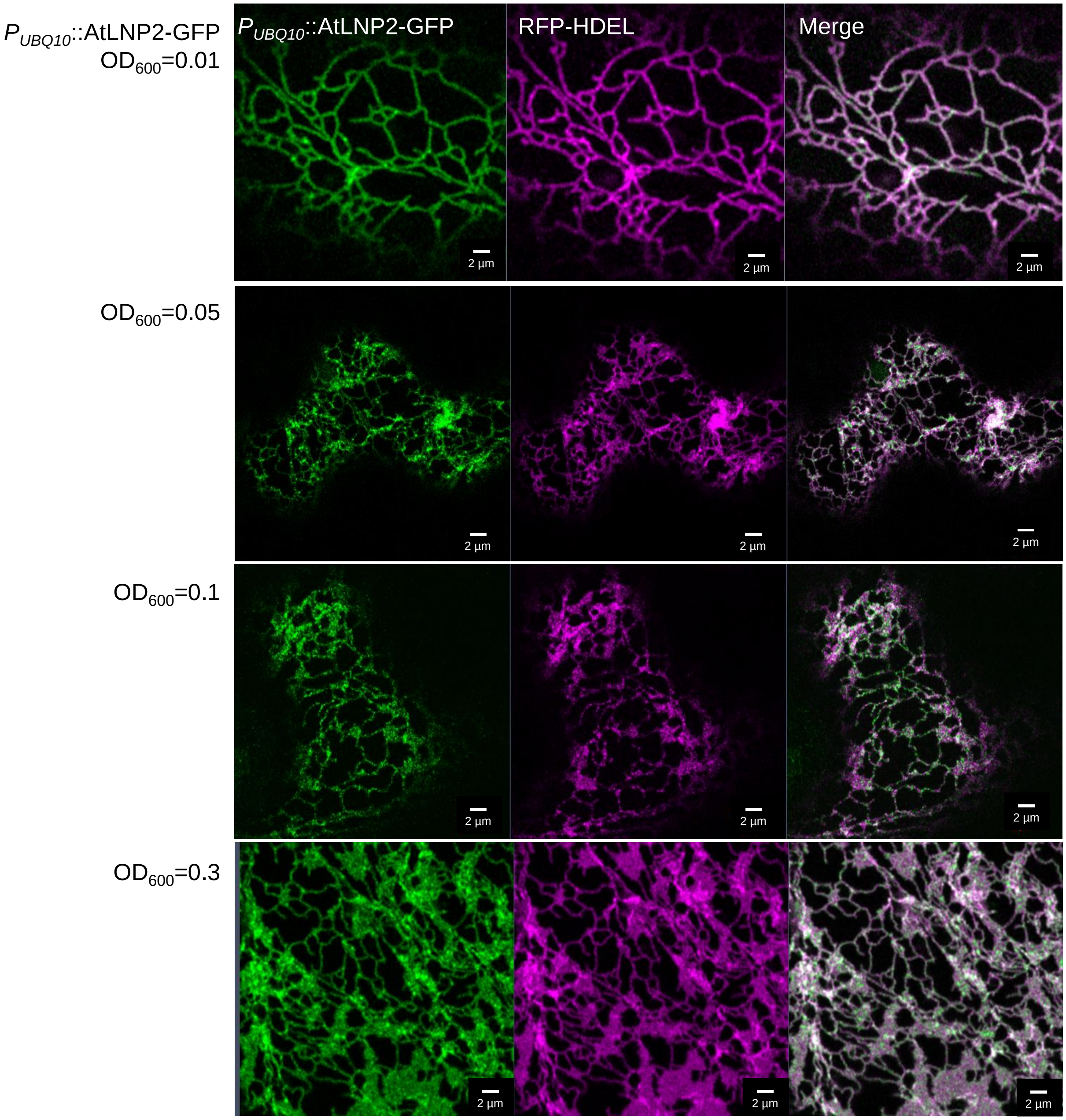

Figure S5: Raw data for protein-protein interactions by FRET-FLIM in tobacco cells.
Raw FRET-FLIM images are shown for the control GFP-RTN1 alone and in combination with RFP-LNP1 or RFP-LNP2, respectively. This analysis takes into account the lifetime values of each pixel within the image visualized by a pseudocolored lifetime map. The graph shows the distribution of lifetimes within the image, with blue shades representing longer GFP fluorescence lifetimes than green ones. Decay curves of a representative single pixel highlight an optimal single exponential fit, where x^2 values from 0.9 to 1.2 were considered an excellent fit to the data points (binning factor of 2). Confocal images for the region of interest showing the GFP construct in green and the mRFP construct in red are included. This specific example shows that RTN1 interacts with both LNP1 and LNP2, because the lifetime values for the GFP/mRFP fusion pair (2.2 ns) are lower than those for the GFP fusion alone (2.4 ns).

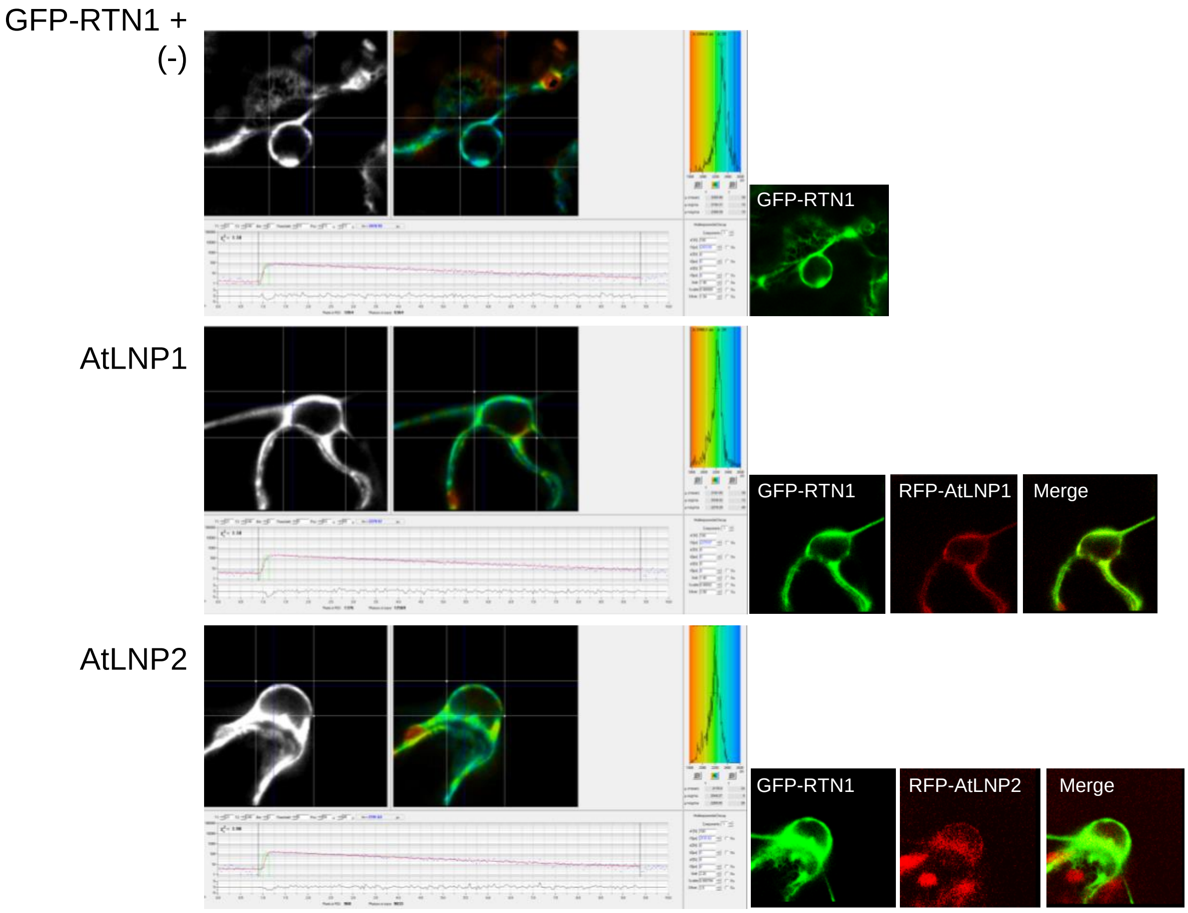

Figure S6: AtLNP1-labelled cisternae display different dynamic behaviours.

P_{UBQ10}::AtLNP1-GFP is transiently expressed in tobacco leaf epidermal cells. Different types of movement dynamics observed in cisternae labelled with P_{UBQ10}::AtLNP1-GFP are listed and shown by representative movies. The total time in seconds [s] of the movie shown is indicated on the right-hand side.

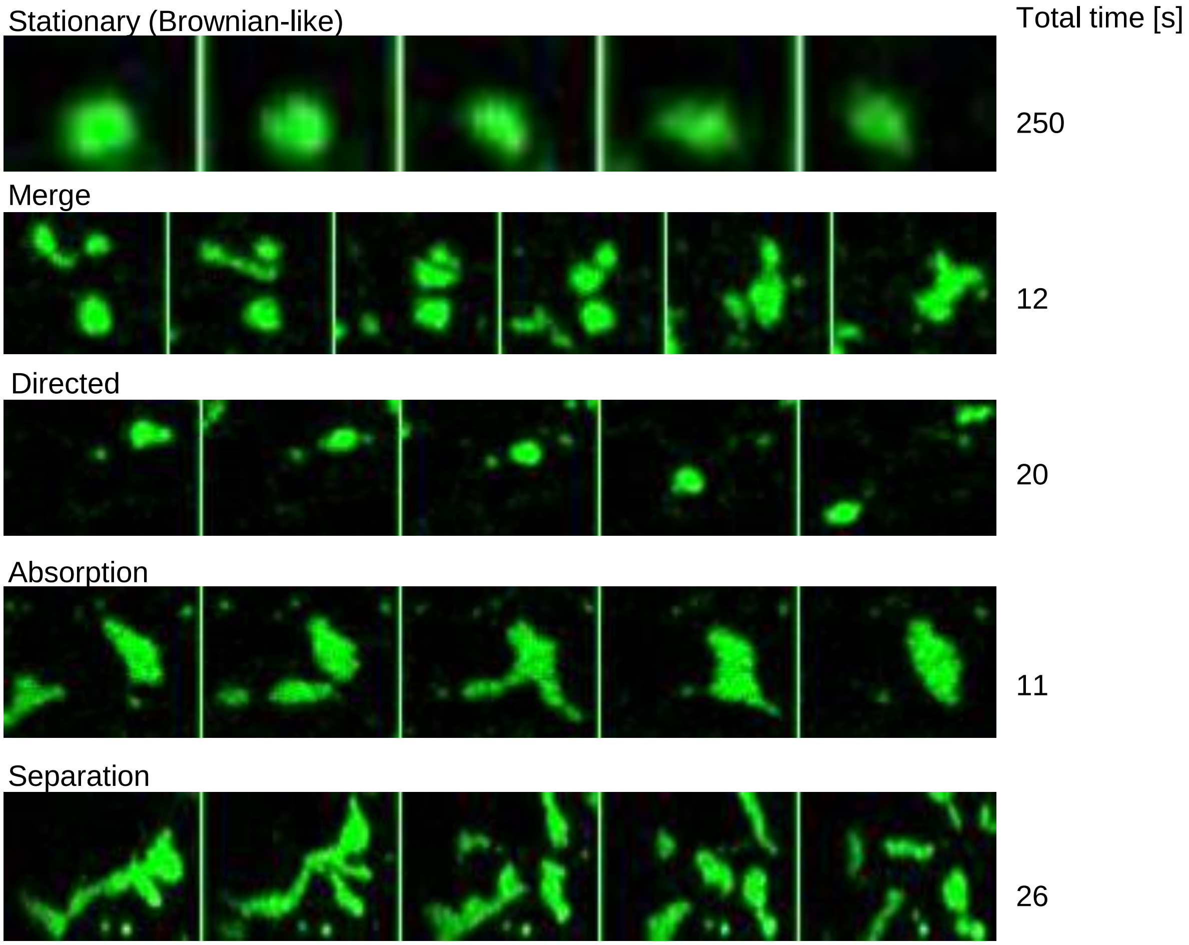

Supplement: Supplementary file 1 — Fig. S1 Analysis of LNP transcript abundance in Arabidopsis amiRNA lines. Fig. S2 Microarray data in the eFP browser for AtLNP1 and AtLNP2. Fig. S3 Lunapark motif analysis in Embryophyta species. Fig. S4 OD expression series for AtLNP1 and AtLNP2 in tobacco epidermal leaf cells (addition to Fig. 4). Fig. S5 Raw data for protein–protein interactions by FRET‐FLIM (addition to Fig. 7). Fig. S6 AtLNP1‐labelled cisternae display different dynamic behaviours. [file NPH-219-990-s001.pdf]
