# Supplementary material for: Arabidopsis Lunapark proteins are involved in ER cisternae formation
Source: New Phytol. 2018 May 25;219(3):990–1004. doi: 10.1111/nph.15228 (PMC6055799; doi:10.1111/nph.15228)
Supplement: Supplementary file 2 — Fig. S7 Example movies for ER network persistency analysis in Fig. 10. [file NPH-219-990-s002.pptx]

## Slide 1
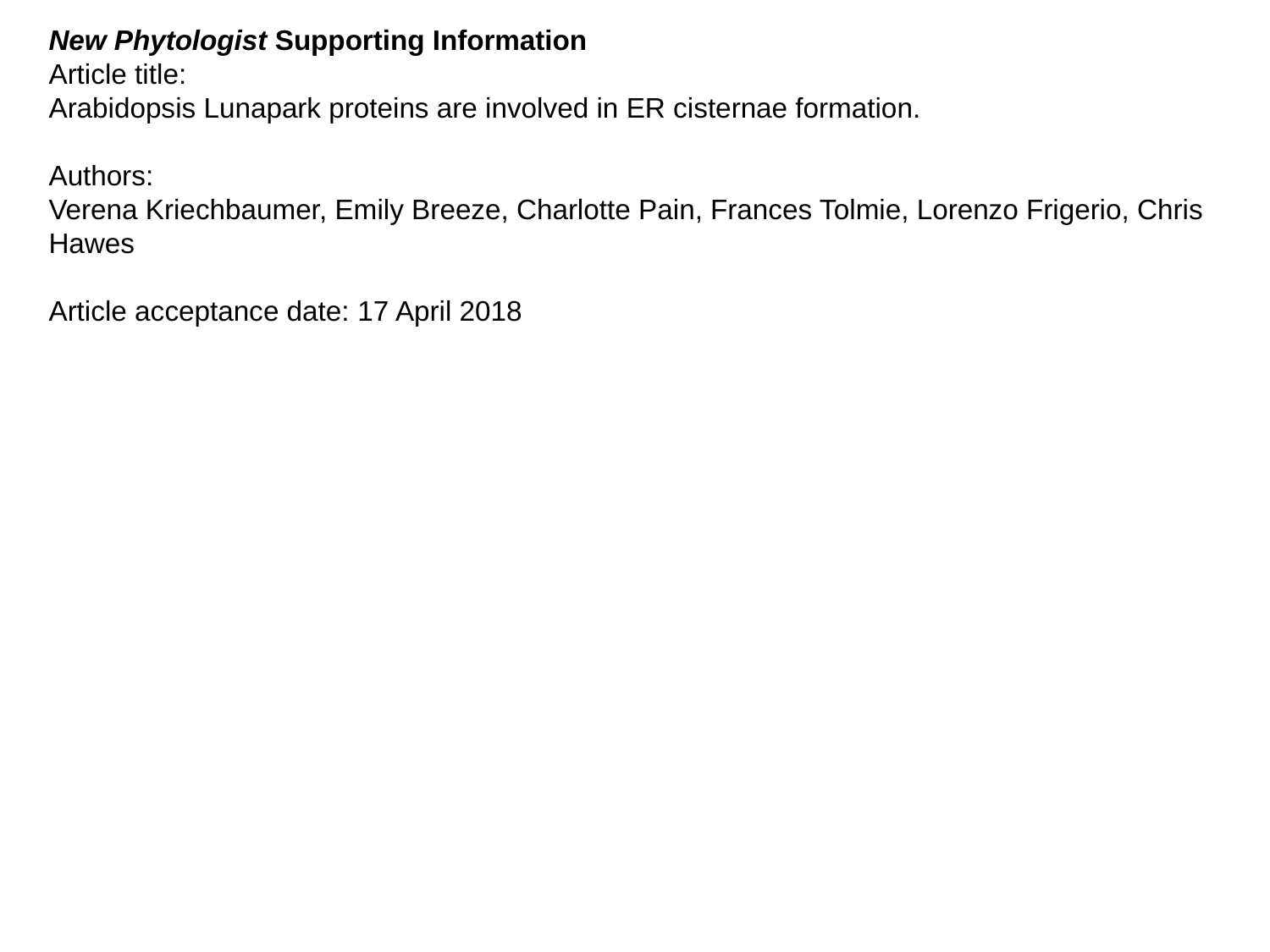

New Phytologist Supporting Information
Article title:
Arabidopsis Lunapark proteins are involved in ER cisternae formation.
Authors:
Verena Kriechbaumer, Emily Breeze, Charlotte Pain, Frances Tolmie, Lorenzo Frigerio, Chris Hawes
Article acceptance date: 17 April 2018

## Slide 2
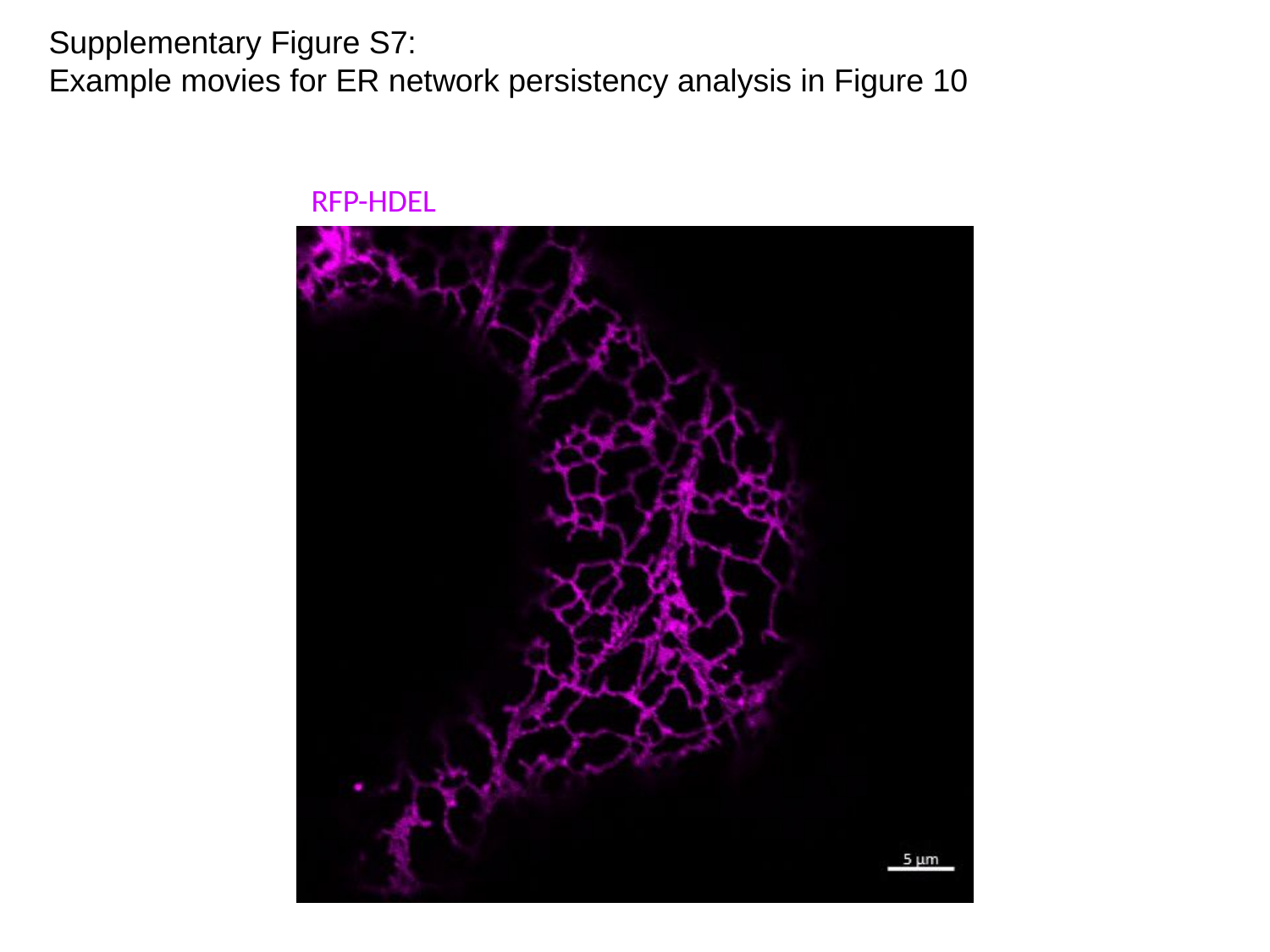

Supplementary Figure S7:
Example movies for ER network persistency analysis in Figure 10
RFP-HDEL

## Slide 3
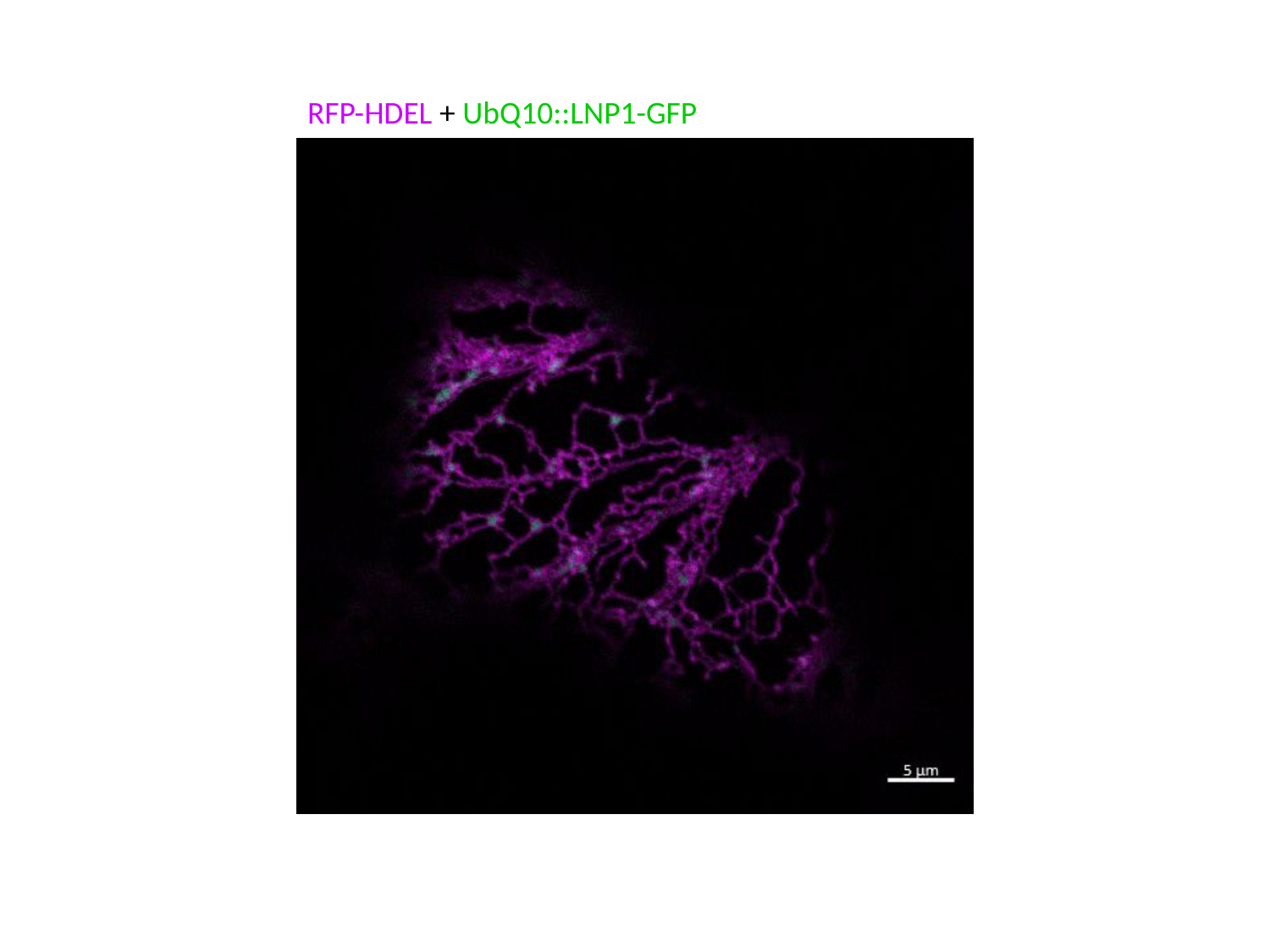

RFP-HDEL + UbQ10::LNP1-GFP

## Slide 4
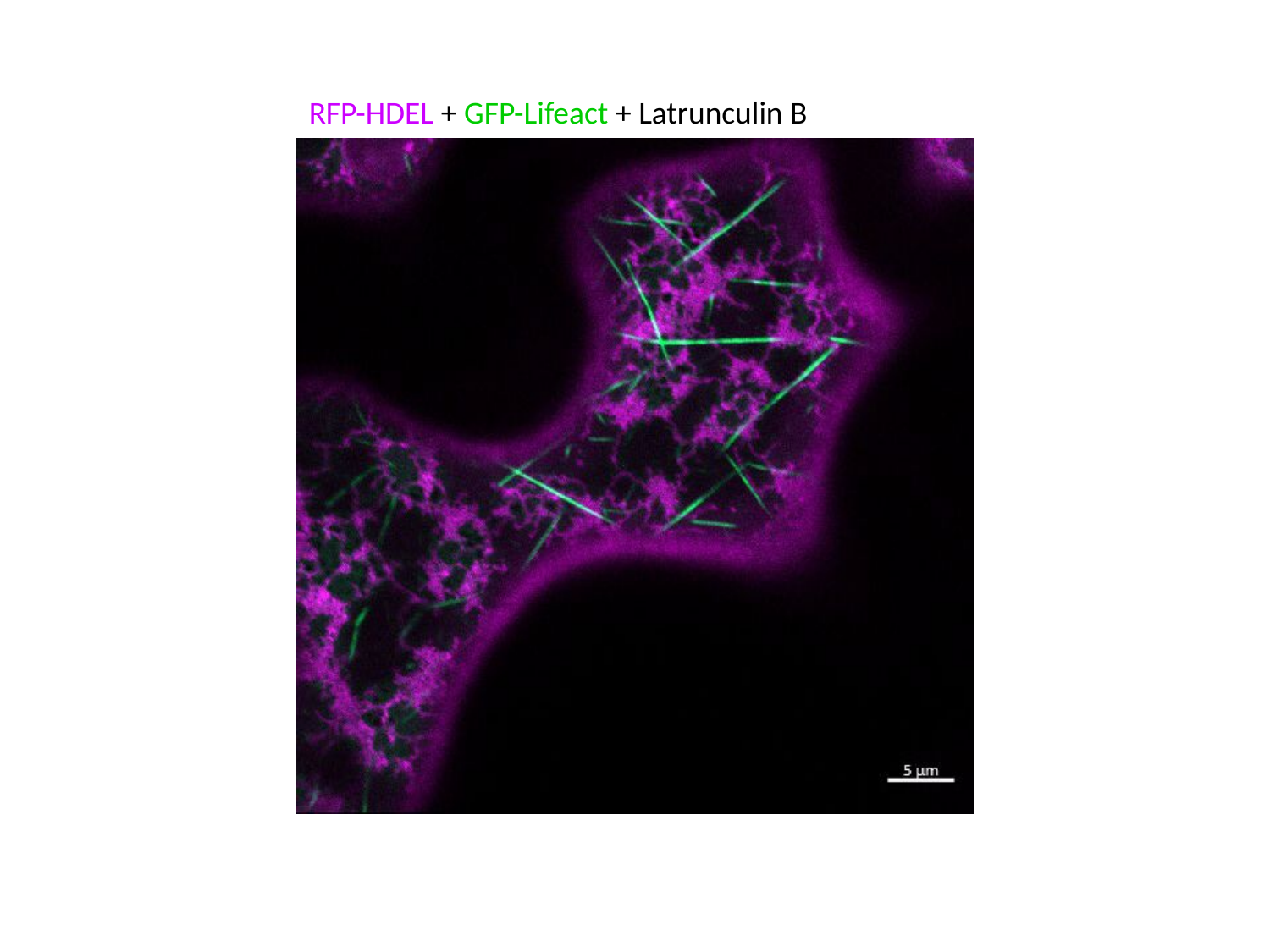

RFP-HDEL + GFP-Lifeact + Latrunculin B
